# Regulation effect of seed priming on sowing rate of direct seeding of rice under salt stress

**DOI:** 10.3389/fpls.2025.1541736

**Published:** 2025-03-06

**Authors:** Yicheng Zhang, Haider Sultan, Asad Shah, Yixue Mu, Yusheng Li, Lin Li, Zheng Huang, Shaokun Song, Ye Tao, Zhenxiang Zhou, Lixiao Nie

**Affiliations:** School of Breeding and Multiplication (Sanya Institute of Breeding and Multiplication), Hainan University, Sanya, China

**Keywords:** direct seeding rice, salt stress, seed priming, sowing rate, grain yield

## Abstract

Direct seeding of rice (DSR) is a widely used method for its labor- and cost-saving advantages. However, the global intensification of soil salinization presents a significant challenge to food security. Increasing sowing rates is a common practice to enhance germination under salt stress, although it leads to higher seed costs. Recently, seed priming has emerged as an effective technique to improve seedling emergence under abiotic stress, but the regulation of seed priming treatment on the sowing rate of DSR under saline soil conditions has rarely been reported. Therefore, field experiments were conducted at two salinity levels of 1.5‰ (1.5 g kg^−1^) (T2) and 3.0‰ (3 g kg^−1^) (T3) and under one non-saline condition (0‰) (T1). The control (P1) consisted of non-primed seeds, while priming treatments included 160 mg L^−^¹ ascorbic acid (P2), γ-aminobutyric acid (P3), and 200 mg L^−^¹ zinc oxide nanoparticles (P4); three sowing rates were applied: 90 (S1), 150 (S2), and 240 seeds m^−2^ (S3). Our results demonstrated that under T1–T3, the germination rate, α-amylase activity, and soluble sugar and protein contents were significantly increased after priming treatments. The contents of reactive oxygen species (i.e., O_2_^−^ and H_2_O_2_) and malondialdehyde (MDA) were decreased, while the activities of enzymatic antioxidants (i.e., superoxide dismutase, peroxidase, and catalase) and the K^+^/Na^+^ ratio of rice were significantly increased after the above seed priming treatments. Under T1–T3, the grain yield increased by 13.39%–36.94% after priming treatments, primarily due to enhanced seed germination, which boosted panicle number per unit area. Among P2–P4 treatments, P4 treatment consistently resulted in the highest yield increase (26.96%–36.94%) compared to P1, outperforming P2 and P3 under T1–T3. Furthermore, under T1–T3, the grain yield with priming treatment at 90 seeds m^−2^ was equivalent to that obtained without priming treatment at 240 seeds m^−2^. The potential mechanisms by which priming treatments enhance rice salt tolerance include increased levels of osmoregulatory substances and elevated activities of antioxidant enzymes, which collectively support improved seed germination. Therefore, to optimize the economic benefits of DSR when the salt concentration is below 3‰, the sowing rate could be reduced to 90 seeds m^−2^ using ZnO-nanoparticle priming treatment.

## 1 Introduction

Rice (*Oryza sativa* L.) is a globally vital crop, serving as a staple food for nearly 50% of the world’s population (Jiang et al., 2023; Yu et al., 2016). With the growing global population, coupled with climate change and environmental stressors such as salinity and drought, food security is facing escalating threats worldwide (Cheeseman, 2016; Wen et al., 2020). Recently, environmental pollution, increased marginal land use, and improper irrigation and land management practices have aggravated soil salinization globally, resulting in substantial declines in crop yields and presenting a significant challenge to agricultural productivity and food security (Wang et al., 2022; Eswar et al., 2021). Therefore, the development and utilization of saline soils for rice cultivation is critical for food security in China and worldwide, especially given the challenges of increasing crop yields and the limited arable land (Mukhopadhyay et al., 2021).

Conventional transplanted rice remains the primary method of rice cultivation in China. As rice cultivation expands, direct seeding of rice (DSR) has become an important cultivation method to address resource and labor shortages (Sandhu et al., 2021). Compared to conventional transplanting, DSR is more susceptible to temperature fluctuations, flooding, and ionic stresses such as sodium (Na^+^) and chloride (Cl^−^), resulting in lower seedling emergence rates and, consequently, reduced yields (Su et al., 2022). Elevated soil salinity inhibits rice seed germination, thereby reducing the germination rate of seeds using DSR (Xu et al., 2017; Shi et al., 2017). A decrease in germination rate reduces the number of rice seedlings, and uneven seedling emergence directly lowers the final rice yield (Chen et al., 2021).

Increasing sowing rates is a conventional practice to ensure a sufficient number of seedlings in the field (Zhang et al., 2023). Excessive sowing rates can lead to an overly dense rice seedling population, which reduces tiller number and significantly lowers grain yield, despite reduced weed growth (Hui, 2018). Moreover, hybrid rice seeds are more expensive than conventionally cultivated rice seeds. Higher sowing rates are often recommended to ensure proper crop establishment under salt stress. As a result, the use of hybrid rice seeds increases costs for farmers, raising the economic burden and reducing profitability in rice production (Sun et al., 2015). However, seed priming has gradually become a sustainable measure to improve seedling emergence in DSR. Seed priming provides resistance and stability to rice seeds (Paul et al., 2022).

It is well known that reactive oxygen species (ROS), including hydrogen peroxide (H_2_O_2_) and superoxide anions (O_2_^·−^), are metabolic by-products generated during normal cellular processes. When crops are exposed to salt stress, ROS accumulate rapidly in plant cells, disrupting the balance between ROS production and their scavenging mechanisms (Madkour, 2019; Zavariyan et al., 2015). ROS accumulation leads to lipid peroxidation, causing malondialdehyde (MDA) accumulation, which is a marker of membrane damage under salt stress (Mushtaq, 2020). To mitigate the harmful effects of ROS, crops have developed antioxidant defense mechanisms, which encompass enzymes such as catalase (CAT), peroxidase (POD), and superoxide dismutase (SOD) (Rajput et al., 2021). There were some studies that demonstrated that seed priming technology could improve the activities of antioxidant enzymes and decrease ROS (H_2_O_2_ and O_2_^·−^) levels, optimizing defense mechanisms during seed germination (Guo et al., 2022).

Previous studies have shown that seed priming technology enhanced the emergence rate of rice seedlings, which accelerated the establishment of the rice population, thereby enhancing root fixation and nutrient absorption (Thakur et al., 2022). Seed priming regulates a range of physiological, biochemical, and molecular processes, enhancing crops’ tolerance to abiotic stresses, promoting faster seedling emergence, and improving overall growth and development (Farooq et al., 2006; Rhaman et al., 2020). Seed priming technology has been demonstrated to enhance crop growth and development, with associated improvements in agronomic traits such as increased plant height, leaf area, tiller number, and biomass (Ma et al., 2018). Additionally, research has indicated that seed priming could regulate the absorption of Na^+^ and K^+^, maintaining ion homeostasis under salt stress and thereby mitigating the adverse effects of salt stress on rice (Iqbal and Ashraf, 2010).

Key factors influencing rice yield include seedling establishment and quality (Gupta et al., 2022). After priming, rice seeds exhibit strong seedling quality, rapid emergence, and high seedling rates, all of which contribute to increased yield at harvest (Ali et al., 2020). Both increased sowing rates and seed priming increase germination rates and seedling quality under salt conditions, benefiting early seedling establishment and providing a strong physiological foundation (Farooq et al., 2019). Moreover, seed priming techniques have also been proven to reduce the need for high seed sowing rates (Farooq, 2011). Therefore, seed priming technology is a practical and sustainable measure to improve rice emergence and reduce seed costs for DSR under saline conditions. While most seed priming studies have been conducted in pot experiments, few have examined the effects of seed priming on rice growth and the regulation effect on the sowing rate of DSR under salt stress in field settings. Therefore, the objectives of this study were 1) to investigate the influences of seed priming treatments and sowing rates on the growth and yield of DSR under different salt stresses and 2) to specifically examine the role of priming treatments in optimizing sowing rates for DSR cultivated under saline soil conditions.

## 2 Materials and methods

### 2.1 Site description

**Figure 1:**
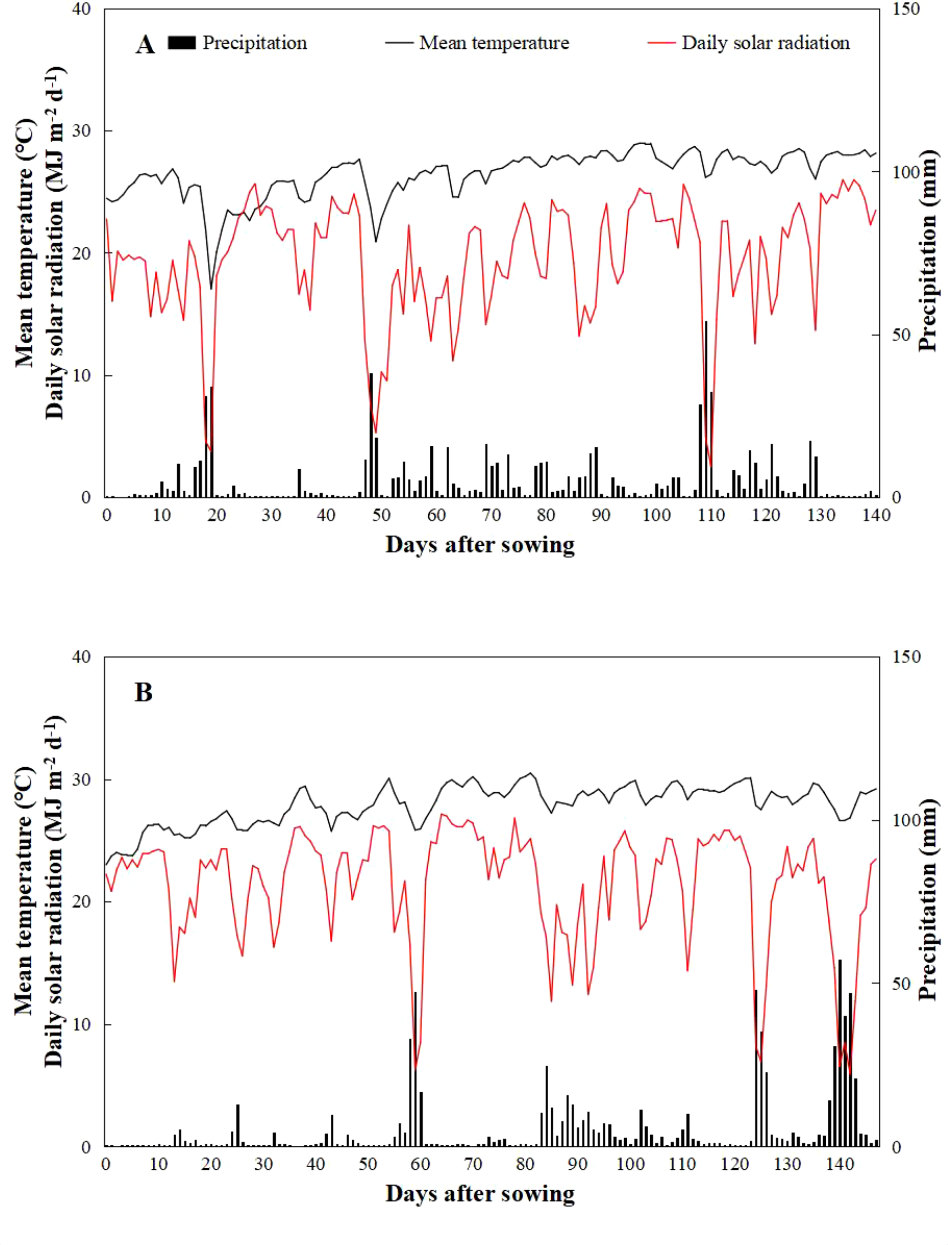
Daily mean temperature, precipitation, and total solar radiation from sowing to maturity of rice in experimental fields of Yazhou District **(A)**, Sanya City, Hainan Province, China, in 2022 and Ledong Li Autonomous County in 2023 **(B)**.

The experiments were carried out in Dadan Village, Yacheng Township, Yazhou District, Sanya City, Hainan Province, China (18°36′N, 109°15′E) and Leyi Village, Jiusuo Township, Ledong Lizu Autonomous County, China (18°44′N, 108°89′E), in 2022 and 2023, respectively. The experimental sites were situated in proximity to the sea and equipped with a water diversion irrigation system suitable for large-scale saline-tolerant rice cultivation. The system pumps seawater and underground freshwater through pipelines and mixes them proportionally in a distribution tank to meet the saline water demand for irrigation of experimental fields. The average temperature, precipitation, and total solar radiation in 2022 were lower than those in 2023 (**Figure 1**). Specifically, the maximum, minimum, and average temperatures recorded in 2022 were 29.11°C, 24.32°C, and 26.43°C, respectively, while in 2023, these values were 30.19°C, 26.04°C°, and 27.88°C, respectively. The average precipitation in 2022 and 2023 were 741.41 mm and 761.93 mm, respectively. The total solar radiation in 2022 was 2,749.10 MJ m^−2^, representing a 14.74% decrease compared to 2023, which recorded 3,154.29 MJ m^−2^. The chemical properties of the soil in the two-season experimental field are detailed in **Table 1**. In 2022, the soil pH, organic matter content, total nitrogen, available phosphorus, and available potassium were 7.61, 0.31%, 0.25 g kg^−1^, 13.62 mg kg^−1^, and 280.51 mg kg^−1^, respectively. In contrast, in 2023, these values were 6.02, 3.68%, 2.26 g kg^−1^, 80.79 mg kg^−1^, and 213.25 mg kg^−1^, respectively.

**Table 1:** Soil properties of the experimental field in 2022 and 2023.

Year	pH	Organic matter (%)	Total nitrogen (g kg^−1^)	Available phosphorus (mg kg^−1^)	Available potassium (mg kg^−1^)
2022	7.61	0.31	0.25	13.62	280.51
2023	6.02	3.68	2.26	80.79	213.25

### 2.2 Experimental design and treatments

The experiment was laid out in a split –split plot design with four replications. Each plot area was 4 m^2^ with a length of 4 m and a width of 1 m. The cultivars Xiangliangyou900 (XLY900) and Longliangyou506 (LLY506) were adopted in the experiment. Both varieties were saline alkali-tolerant hybrid early rice in this experiment. Three salt concentrations were selected, namely, 0‰ (T1), 1.5‰ (1.5 g kg^−1^) (T2), and 3.0‰ (3 g kg^−1^) (T3). One non-priming treatment (P1) as a control while three priming treatments were applied in the sub-plot: 160 mg L^−1^ ascorbic acid (ASA) priming (P2), 160 mg L^−1^ γ-aminobutyric (GABA) priming (P3), and 200 mg L^−1^ zinc oxide nanoparticles (ZnO-Nano) priming (P4). We previously conducted screening tests on priming treatments and found that under three salinity levels, the best priming effect on the germination rate of rice seeds was observed on 160 mg L^−1^ ASA, 160 mg L^−1^ GABA, and 200 mg L^−1^ ZnO nanoparticles (Mu et al., 2023). Therefore, these three priming treatments were selected for field tests. The above reagents were purchased from Shanghai Biochemical Technology McLean Reagent Co., Ltd. (Shanghai, China), and the nanoparticle size of ZnO-Nano was 30 ± 10 nm. Three sowing rates were added in the sub-plot, namely, 90 seeds m^−2^ (S1), 150 seeds m^−2^ (S2), and 240 seeds m^−2^ (S3), representing low, medium, and high sowing rates, respectively.

In the 2-year field experiment, nitrogen fertilizer was selected as urea (N ≥ 46.0%) with 150 kg N ha^−1^, distributed according to the following proportions: basal fertilizer, tiller fertilizer, and booting fertilizer at a 1:1:1 ratio. Phosphorus fertilizer was applied as calcium superphosphate (P_2_O_5_ ≥ 14.0%) at a rate of 60 kg P_2_O_5_ ha^−1^, entirely as a basal fertilizer. Potassium fertilizer was applied as potassium chloride (KCl) (K_2_O ≥ 60.0%) at a rate of 100 kg K ha^−1^, split between basal and booting fertilizers in a 1:1 ratio. Meticulous management practices were implemented to mitigate potential grain yield loss from pest and disease infestations.

Before the priming treatment, the seeds were placed in a desiccant for dehydration and drying until the moisture content of the seeds was less than 10%. Then, the seeds were stored in an aluminum foil bag under vacuum, with the initial germination rate exceeding 90%. Afterward, the sterilized and dried seeds were placed into a priming solution including the 160 mg L^−1^ ASA priming, the 160 mg L^−1^ GABA priming, and the 200 mg L^−1^ ZnO-Nano priming. The weight of the seed and the volume ratio of the priming solution were 1: 5 (W: V). Among them, the ZnO-Nano priming solution was subjected to ultrasonic dissolution for a duration of 30 minutes. The seeds were incubated at a constant temperature of 25°C under dark conditions for 24 hours, and the priming solution was replaced once after 12 hours of priming. Following 24 hours of priming treatments, the seeds were rinsed three times with distilled water, and the surface water was removed. The seeds were then placed in a blast drying oven at 25°C until their total weight was equivalent to that prior to priming. At the conclusion of the priming treatment, seeds were woven into the sowing belt using a seed weaving machine (SH-BZ-III, Shandong Jiesheng Heavy Machinery Co., Ltd., Jining, China). Strip seeding used wet direct sowing, and the sowing date of both experimental years was March. Before sowing, a rotary tiller was used to rotate the ground to wash salt two to three times until the soil salt content of the field was reduced to below 2‰. The excess water on the soil surface was drained, seeds were sowed when there was no bright water in the field, and row spacing was carried out at 20 cm. Saline irrigation was implemented continuously from sowing to harvest. The salinity concentration was monitored daily using an electrical conductivity meter, and freshwater or seawater was promptly supplemented as needed. This approach ensured the continuity and stability of saline stress throughout the experimental period. In the first week after sowing, the plots were maintained under a moist condition to promote optimal seedling establishment. Subsequently, shallow water irrigation with the appropriate salinity was applied until the five-leaf stage. Following this, fields were flooded with the corresponding saline water, and the water level was maintained at 5–10 cm until 2 weeks before harvest.

### 2.3 Data recorded

#### 2.3.1 Meteorological data and determination of soil chemical properties

Soil samples were collected before sowing from each experimental site at a depth of 0–20 cm with the help of an auger. Each sample comprised a composite of three randomly selected cores from each plot. The fresh soil samples were thoroughly mixed, while a portion of the composite sample was air-dried and subsequently sieved through a 1-mm mesh. After sieving, the soil samples were kept in clean plastic bags for initial soil analysis. The pH of the soil, along with its organic matter content, total nitrogen, available phosphorus, and available potassium, was analyzed. The average temperature, precipitation, and solar radiation were recorded using a micro-meteorological station. The micro-meteorological station was installed 500 m away from the test field. The micro-meteorological station was equipped with various specialized instruments, including an air temperature and humidity sensor, a data logger, a rain gauge, total solar radiation sensors, and wind speed and direction sensors.

#### 2.3.2 Determination of seed germination

In the 2-year field experiment, the seed germination percentage was monitored daily in accordance with the methodology described by AOSA (1990). Seeds were considered germinated when the radicle length exceeded 2 mm. The number of seedlings within 1 m^2^ in each plot was investigated 5 days after sowing to calculate the emergence rate. The investigation interval was 2 days, and the number of examinations was seven times. The germination rate was measured by the following formula: the germination rate (%) = (number of emerged seeds on a given day/total number of seeds tested) * 100.

#### 2.3.3 Determination of α-amylase activity, osmoregulatory substances, ROS levels, and antioxidant enzymes

Seven days after sowing, seedlings were sampled, washed with double-distilled water, surface-dried using blotting paper, and then immediately stored in liquid nitrogen before being transferred to a −80°C freezer for subsequent analysis. Approximately 0.1– 0.2 g rice seedlings were weighed to quantify α- amylase activity, osmoregulatory substances, ROS levels, and antioxidant enzymes. The kits for testing soluble sugar content, proline content, O_2_^•−^, POD, CAT, and H_2_O_2_ were obtained from Beijing Solarbio Science & Technology Co., Ltd. (Beijing, China), while the kits for analyzing α- amylase activity, SOD, and MDA were obtained from Nanjing Jiancheng Bioengineering Institute (Nanjing, China) (Mu et al., 2023).

#### 2.3.4 Determination of tiller or panicle number and aboveground biomass

Throughout the 2-year experiment, one row of 1-m length (0.2 m^2^) was sampled at the mid-tillering stage (MT), panicle initiation stage (PI), heading stage (HD), and physiological maturity stage (PM) to investigate the tiller or panicle number, and then plant samples were divided into straw and panicles for further plant analysis. After drying the plants at 105°C for 15 minutes, the aboveground biomass of each plot was measured by oven-drying at 85°C for 72 h until a constant weight was achieved.

#### 2.3.5 Analysis of grain yield and its components

The grain yield was measured from a 1-m^2^ sampling area located at the center of each plot and standardized to a moisture content of 0.14 g H_2_O g^−1^. Subsequently, a 1-m-long row of plants (0.2 m^2^) was sampled at each plot to assess additional yield components. Panicle numbers in the sampled rows were counted in order to calculate the panicle number per square meter. The spikelets were obtained through manual threshing, followed by separation into filled and unfilled spikelets by employing the water selection method. After air-drying, unfilled spikelets were distinguished from the half-filled spikelets by winnowing. The total air-dried mass of the filled and unfilled spikelets was weighed, and small samples were taken for analysis: three subsamples of filled spikelets (30 g), three subsamples of unfilled spikelets (2 g), and all the half-filled spikelets were counted to calculate the yield components, including the number of effective panicles per unit area, the number of spikelets per panicle, the filled grain rate, and the 1000-grain weight.

#### 2.3.6 Sodium (Na^+^) and potassium (K^+^) estimation

The dried samples of rice straw were ground into powder. Subsequently, 0.1 g of the crushed sample was accurately weighed from each treatment, and the samples were digested using the H_2_SO_4_·H_2_O_2_ method and concentrated in a microwave oven. The concentration of Na^+^ and K^+^ at the PM stage was measured using a flame photometer, and the K^+^/Na^+^ ratio was calculated (Li et al., 2023).

### 2.4 Statistical analysis

In the present study, due to the consistent responses of two rice varieties adopted in all treatments, the mean value of both varieties was present for all data in order to reduce the length of the text (**Supplementary Figures S1**–**S9**; **Supplementary Tables S1**, **S2**). The data were analyzed using analysis of variance (ANOVA) using the Statistix 9.0 software. The differences between treatments were compared using the least significance difference (LSD) test at the 0.05, 0.01, and 0.001 probability levels. Graphical representations of the data were generated using Origin 2021.

## 3 Results

### 3.1 Effects of priming on seed germination of DSR under salt stress

**Figure 2:**
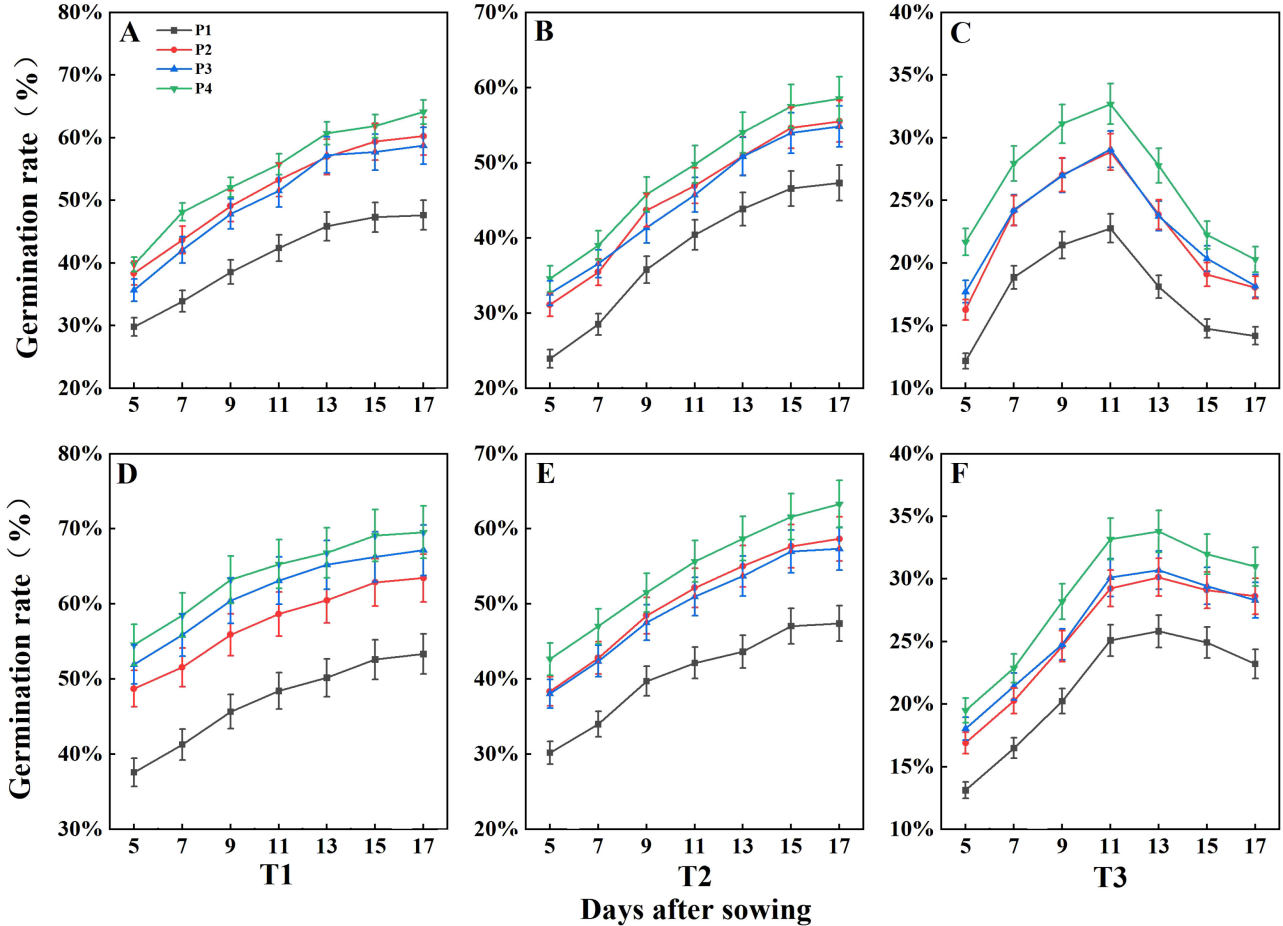
Effects of seed priming treatments on germination rate of direct seeding of rice under salt stress in 2022 **(A–C)** and 2023 **(D–F)**. Error bars represent the standard error. T1, T2, and T3 represent salinity levels of 0‰, 1.5‰, and 3‰, respectively. P1, P2, P3, and P4 represent no-priming treatment, ASA_160mg/L_ priming treatment, GABA_160mg/L_ priming treatment, and ZnO-Nano_200mg/L_ priming treatment, respectively.

The seed germination of DSR was negatively impacted by increasing salinity levels, whereas all three priming treatments enhanced seed germination across the three salinity conditions (**Figure 2**). Under the non-saline condition (T1), the average germination rates for rice seeds during 2 years were 50.49%, 61.84%, 62.94%, and 66.82% for treatments P1, P2, P3, and P4, respectively. Under the T2 condition, seed germination for rice was 47.35% under the non-primed (P1) treatment, with germination rates for P2, P3, and P4 showing increases of 9.75%, 8.75%, and 13.56%, respectively, compared to P1 during 2 years. Under the T3 condition, the germination rate was 18.70% in P1, while the rates for P2, P3, and P4 were enhanced by 4.63%, 4.53%, and 6.93%, respectively, compared to P1. The above results suggested that the P4 treatment performed best among the priming treatments under all three salinity conditions. As shown in **Figure 2**, germination rates under T1 and T2 remained stable over time. However, under T3, the germination rate peaked between days 11 and 13 before declining. This suggests that the higher salt concentration in T3 prolonged exposure to elevated salinity levels, ultimately leading to a reduction in germination over time.

### 3.2 Effects of priming on α-amylase activity and contents of osmoregulatory substances under salt stress

**Figure 3:**
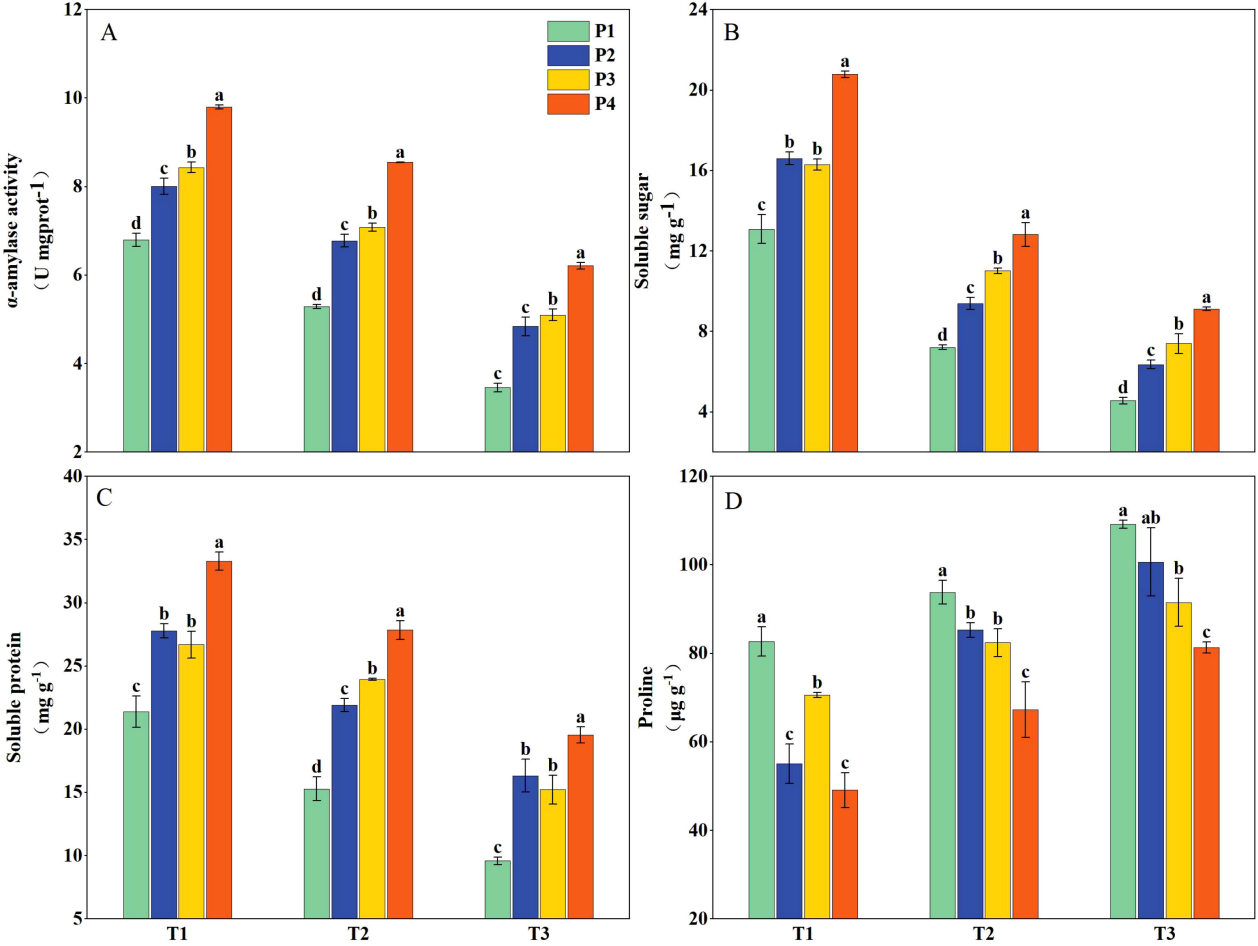
The α-amylase activity **(A)** and concentration of soluble sugar **(B)**, soluble protein **(C)**, and proline **(D)** of primed and non-primed seeds under different salt stress at 7 days after sowing (average of two varieties). Within a column, means followed by the same letter are not significantly different at the 0.05 probability level according to the least significant difference test (LSD 0.05). T1, T2, and T3 represent salinity levels of 0‰, 1.5‰, and 3‰, respectively. P1, P2, P3, and P4 represent no-priming treatment, ASA_160mg/L_ priming treatment, GABA_160mg/L_ priming treatment, and ZnO-Nano_200mg/L_ priming treatment, respectively.

Priming treatments significantly influenced α-amylase activity, soluble sugar content, soluble protein content, and proline levels in rice seedlings subjected to salt stress (**Figure 3**). Under T1, the α-amylase activity, soluble sugar content, and soluble protein content had average increases of 28.66%, 36.70%, and 36.69%, respectively, while the proline content decreased by 29.57% across all priming treatments (P2, P3, and P4) compared to P1. Under T2, the above-mentioned parameters had average decreases of 16.22%, 39.42%, and 18.47%, respectively, and the proline content increased by 27.71% compared to T1. Following priming treatments, the above-mentioned parameters showed increases of 41.29%, 53.51%, and 60.68%, respectively, and the proline content decreased by 16.50% compared with P1. Under T3, the above-mentioned parameters decreased by 40.68%, 58.90%, and 44.38%, respectively, and proline content increased by 48.63% compared to T1. However, with all priming treatments, the above-mentioned parameters increased by 55.68%, 67.16%, and 77.47%, respectively, and proline content decreased by 16.52% compared to P1. Additionally, across all salinity levels, the P4 priming treatment consistently resulted in significantly higher α-amylase activity, soluble sugar content, and protein content and lower proline content compared to P2 and P3 (**Figure 3**).

### 3.3 Effects of priming on ROS levels and antioxidant enzymes under salt stress

**Figure 4:**
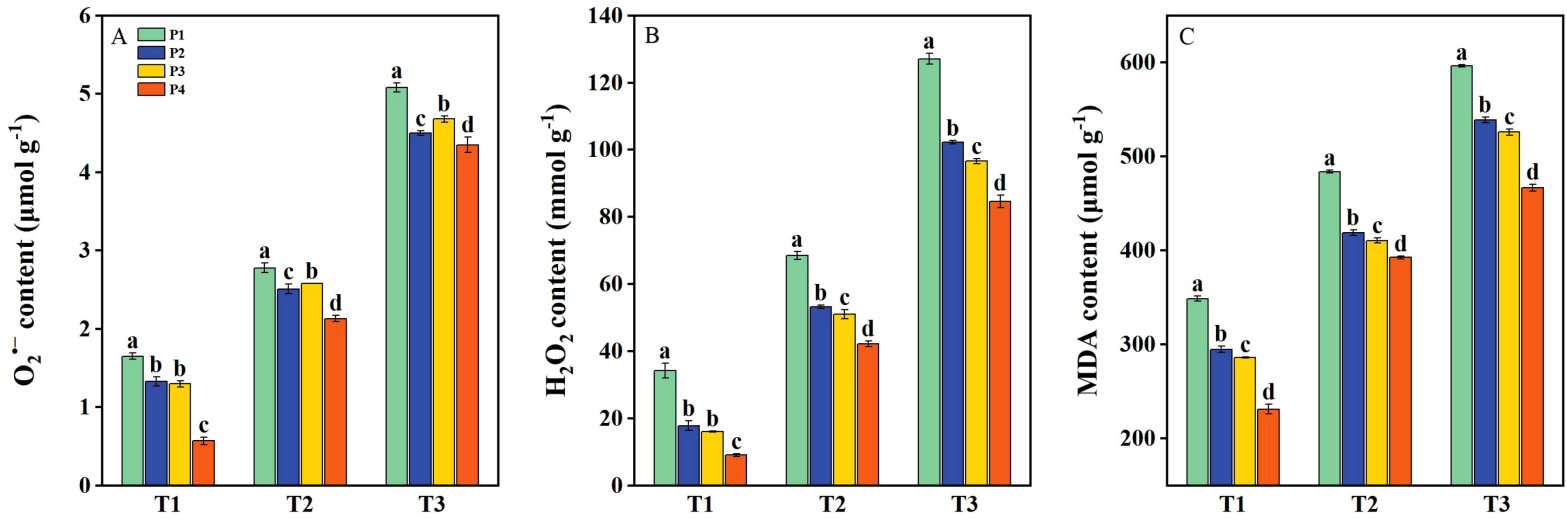
The concentration of O_2_^•−^ **(A)**, H_2_O_2_ **(B)**, and malondialdehyde (MDA) **(C)** of primed and non-primed seeds under different salt stress at 7 days after sowing (average of two varieties). Note: Within a column, means followed by the same letter are not significantly different at the 0.05 probability level according to the least significant difference test (LSD 0.05). T1, T2, and T3 represent salinity levels of 0‰, 1.5‰, and 3‰, respectively. P1, P2, P3, and P4 represent no-priming treatment, ASA_160mg/L_ priming treatment, GABA_160mg/L_ priming treatment, and ZnO-Nano_200mg/L_ priming treatment, respectively.

The O_2_^•−^, H_2_O_2_, and MDA contents were significantly increased under salt stress (T2 and T3) (**Figure 4**), while the ROS content in rice plants was markedly reduced following priming treatments. Under T1, the O_2_^•−^, H_2_O_2_, and MDA contents had average decreases of 35.26%, 58.10%, and 22.40%, respectively, across all priming treatments compared to P1. Under T2, the O_2_^•−^, H_2_O_2_, and MDA contents had average decreases of 13.69%, 28.80%, and 15.81%, respectively, under priming treatments compared to P1. Similarly, under T3, the reductions were 11.19%, 25.60%, and 14.43%, respectively, across priming treatments compared to P1. Under all three salinity levels, the P4 treatment resulted in the most substantial reduction of ROS accumulation compared to P2 and P3 (**Figure 4**).

**Figure 5:**
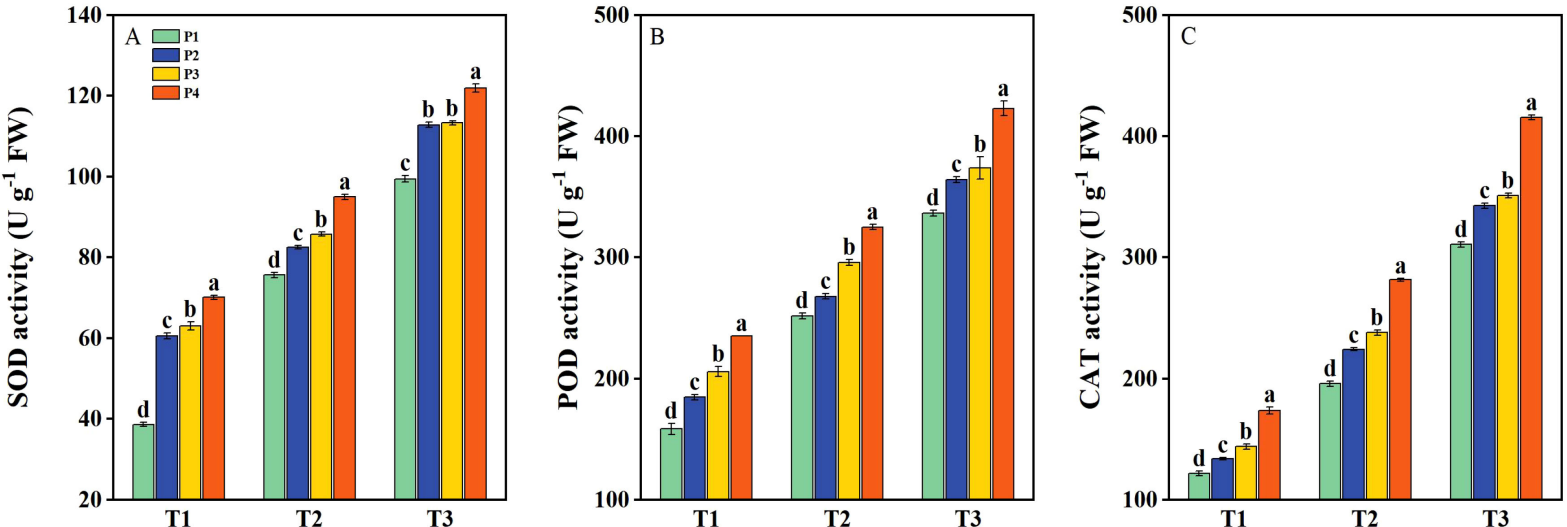
The activity of superoxide dismutase (SOD) **(A)**, peroxidase (POD) **(B)**, and catalase (CAT) **(C)** of primed and non-primed seeds under different salt stress at 7 days after sowing (average of two varieties). Within a column, means followed by the same letter are not significantly different at the 0.05 probability level according to the least significant difference test (LSD 0.05). T1, T2, and T3 represent salinity levels of 0‰, 1.5‰, and 3‰, respectively. P1, P2, P3, and P4 represent no-priming treatment, ASA_160mg/L_ priming treatment, GABA_160mg/L_ priming treatment, and ZnO-Nano_200mg/L_ priming treatment, respectively.

The activities of SOD, POD, and CAT in rice seedlings were significantly enhanced under salt stress and further improved by priming treatments (**Figure 5**). Under T1, the activities of POD, SOD, and CAT had average increases of 31.62%, 67.14%, and 23.69%, respectively, with priming treatments compared to P1. Under T2, the above-mentioned antioxidant enzymes had average increases of 17.75%, 15.98%, and 26.56%, respectively, following priming treatments compared with P1. Under T3, the above-mentioned antioxidant enzymes had average increases of 15.05%, 16.68%, and 19.05%, respectively, across priming treatments compared to P1. Overall, under all three salinity levels, the P4 treatment consistently resulted in the highest increases in the activities of SOD, POD, and CAT compared to P2 and P3 (**Figure 5**).

### 3.4 Effects of priming and sowing rates on tiller number and aboveground biomass under salt stress

**Figure 6:**
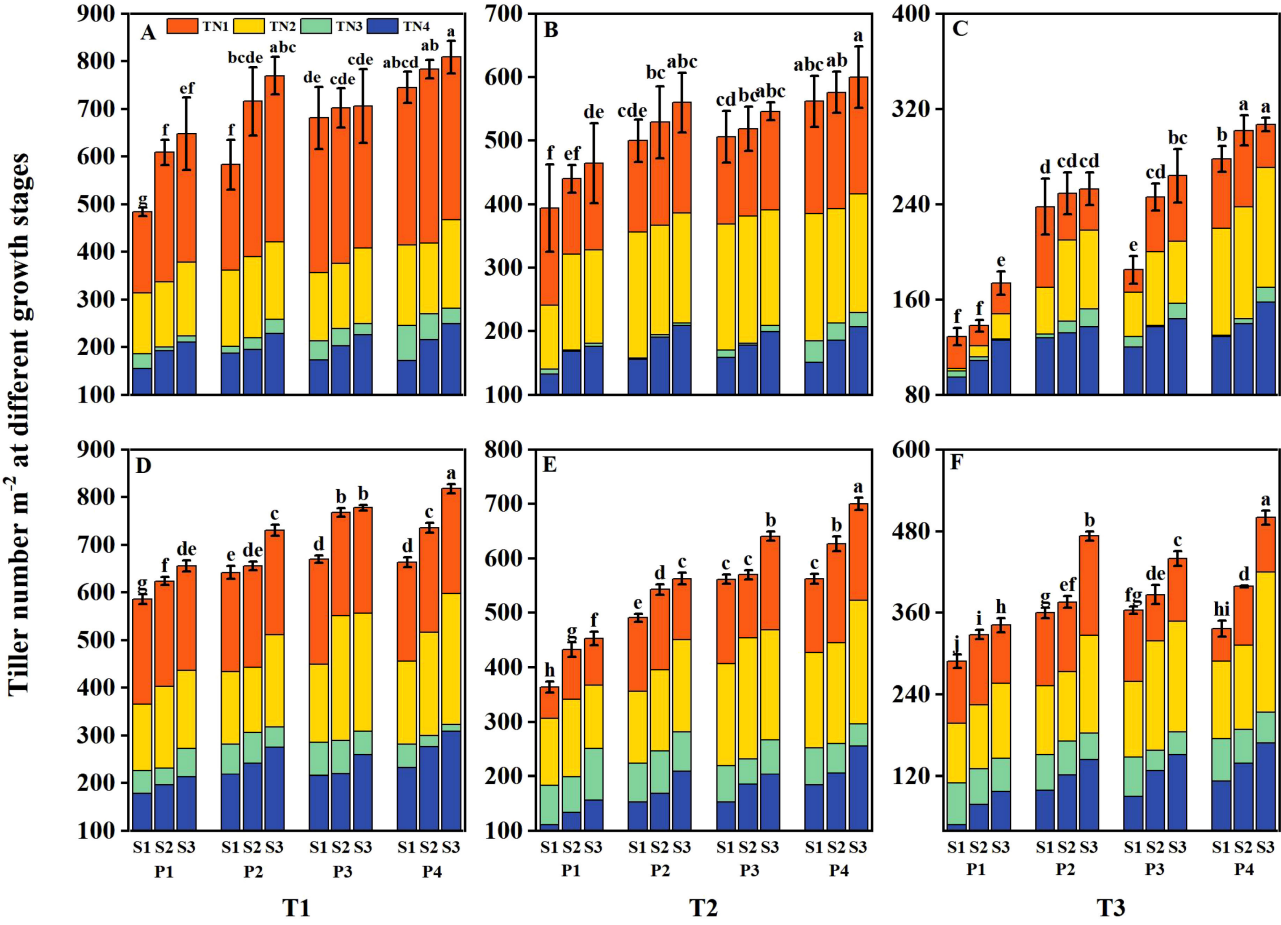
Effects of priming treatments and sowing rates on tiller number at different growth stages in 2022 **(A–C)** and 2023 **(D–F)** (average of two varieties). Within a column, means followed by the same letter are not significantly different at the 0.05 probability level according to the least significant difference test (LSD 0.05). TN1 represents tiller number at the mid-tillering stage, TN2 represents tiller number at the panicle initiation stage, TN3 represents tiller number at the heading stage, and TN4 represents tiller number at the physiological maturity stage. T1, T2, and T3 represent salinity levels of 0‰, 1.5‰, and 3‰, respectively. P1, P2, P3, and P4 represent no-priming treatment, ASA_160mg/L_ priming treatment, GABA_160mg/L_ priming treatment, and ZnO-Nano_200mg/L_ priming treatment, respectively. S1, S2, and S3 represent three sowing rates (90, 150, and 240 seeds m^−2^, respectively).

**Figure 7:**
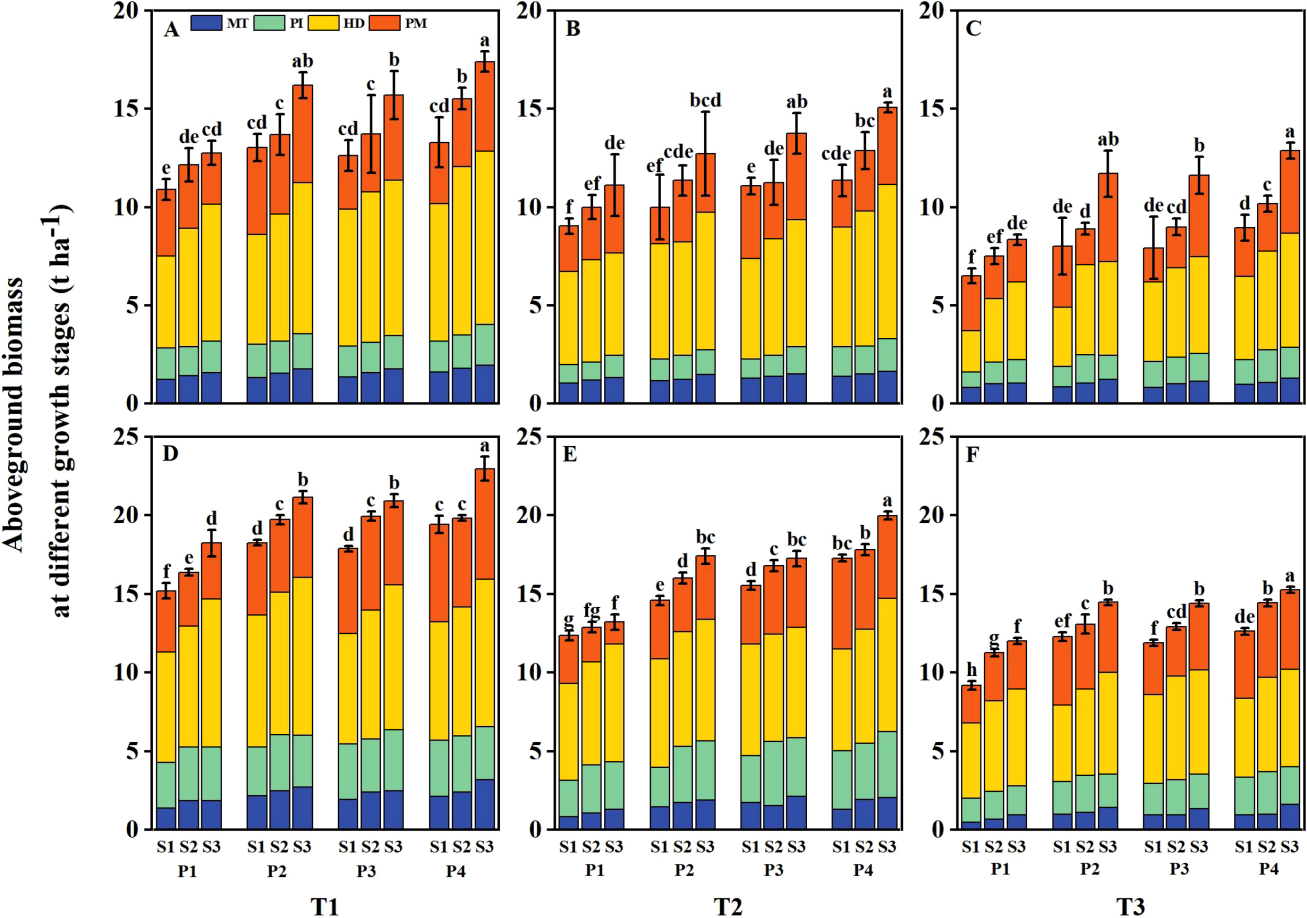
Effects of priming treatments and sowing rates on aboveground biomass at different growth stages in 2022 **(A–C)** and 2023 **(D–F)** (average of two varieties). Different lowercase letters represent a significant difference at the maturity stage at 0.05 levels according to the least significance difference (LSD) test. MT represents the mid-tillering stage, PI represents the panicle initiation stage, HD represents the heading stage, and PM represents the physiological maturity stage. T1, T2, and T3 represent salinity levels of 0‰, 1.5‰, and 3‰, respectively. P1, P2, P3, and P4 represent no-priming treatment, ASA_160mg/L_ priming treatment, GABA_160mg/L_ priming treatment, and ZnO-Nano_200mg/L_ priming treatment, respectively. S1, S2, and S3 represent three sowing rates (90, 150, and 240 seeds m^−2^, respectively).

The tiller number and aboveground biomass of DSR were reduced at various growth stages under salt stress (T2 and T3), with the reduction severity increasing alongside higher salt concentrations (**Figures 6**, **7**). However, both parameters were significantly enhanced under different priming treatments and sowing rate levels. Under T1, the tiller number and aboveground biomass of DSR increased by 13.56%– 18.69%, 13.39%– 19.30%, and 26.37%– 28.67% and by 13.47%– 29.13%, 13.20%– 23.95%, and 19.71%– 40.45% under P2, P3, and P4, respectively, compared to P1 during 2 years. Under T2, the above-mentioned parameters decreased by 13.84%– 29.45% and 12.37%– 29.78%, respectively, compared to T1. However, following P2, P3, and P4 treatments, the above-mentioned parameters increased by 16.80%– 25.11%, 13.33%– 31.36%, and 27.20%– 42.57%, and 17.66%– 33.33%, 16.31%– 39.11%, and 28.70%– 46.67%, respectively, compared to P1. Under T3, the above-mentioned parameters were reduced 39.20%– 60.73% and 36.02%– 53.78%, respectively, compared to T1, However, after P2, P3, and P4 treatments, the above-mentioned parameters increased by 23.60%– 38.40%, 21.60%– 42.98%, and 36.00%– 67.05%, and 17.53%– 65.06%, 23.59%– 26.03%, and 30.63%– 76.51%, respectively, compared to P1. Across all salinity levels and priming treatments, both the tiller number and aboveground biomass increased with higher sowing rates. However, the tiller number and aboveground biomass in the P2S1, P3S1, and P4S1 treatments were not significantly different from those in the P1S3 treatment during 2 years.

### 3.5 Effects of priming and sowing rates on grain yield and its components of rice under salt stress

The grain yield of DSR was decreased under salt stress (T2 and T3), with greater reductions observed at higher salinity levels (**Tables 2**, **3**), while grain yield was increased under priming and sowing rate treatments primarily due to the increased panicle number. Under T1, the grain yield increased by 14.40%, 13.39%, and 26.96% under P2, P3, and P4 treatments, respectively, compared with P1 during 2 years. Under T2, the grain yield decreased by 28.76% compared to T1, while under P2, P3, and P4 treatments, the grain yield increased by 15.33%, 14.41%, and 29.35%, respectively, compared with P1. Under T3, the grain yield decreased by 70.75% compared with T1, while under P2, P3, and P4 treatments, it increased by 25.76%, 23.38%, and 36.94%, respectively, compared with P1. The above results suggested that under all three salinity levels, the grain yield under the ZnO-nanoparticle priming (P4) treatment was the highest in the three priming treatments. Furthermore, under all three salinity levels, the grain yield was increased with higher sowing rates, peaking at S3. No significant difference was observed in grain yield among three sowing rates within the same priming treatment in 2022, while significant differences were observed in 2023. Under T1, the grain yield had average increases of 9.18% and 20.05% under S2 and S3, respectively, compared with S1 during 2 years. Under T2, the grain yield had average increases of 5.91% and 17.36% under S2 and S3, respectively, compared with S1. Under T3, the grain yield had average increases of 16.61% and 33.87% under S2 and S3, respectively, compared with S1. Additionally, across all salinity levels, grain yield under the P2S1, P3S1, and P4S1 treatments was not significantly different from the P1S3 treatment in both 2022 and 2023.

**Table 2:** Effects of priming treatments and sowing rates on grain yield and its components in rice under salt stress in 2022 (average of two varieties).

Salinity	Priming treatment	Sowing rate	Yield (t ha^−1^)	Panicles (no. m^−2^)	Spikelets panicle^−1^	Filled grains (%)	1000-grain weight (g)
T1	P1	S1	3.49f	155f	125a	63.33a	22.37d
		S2	3.84def	193cde	121a	59.75a	23.01c
		S3	4.31abcde	211bcd	134a	64.67a	23.23bc
	P2	S1	3.74def	187def	134a	61.67a	23.55abc
		S2	4.16bcdef	195bcde	140a	60.33a	23.46abc
		S3	4.82ab	229ab	136a	65.33a	23.58ab
	P3	S1	3.69ef	173ef	152a	61.75a	23.41abc
		S2	4.10cdef	203bcde	136a	64.00a	23.82a
		S3	4.61abc	226abc	154a	66.33a	23.75ab
	P4	S1	4.23abcde	172ef	139a	62.75a	23.43abc
		S2	4.36abcd	216abcd	132a	59.50a	23.50abc
		S3	4.85a	249a	136a	63.67a	23.25bc
		Mean	4.18A	200A	137A	62.61A	23.37A
T2	P1	S1	3.00d	133f	122a	58.44a	21.31c
		S2	3.00d	167bcdef	111a	57.36a	21.58abc
		S3	3.47bcd	175abcde	116a	59.70a	21.69abc
	P2	S1	3.36cd	156def	135a	63.77a	22.25ab
		S2	3.57bcd	190abc	136a	57.76a	21.81abc
		S3	3.95abc	207a	113a	62.57a	21.99abc
	P3	S1	3.44bcd	159cdef	145a	62.87a	21.68abc
		S2	3.76abc	178abcde	129a	61.67a	22.09abc
		S3	4.05ab	199ab	122a	59.31a	22.38a
	P4	S1	3.93abc	151ef	136a	60.77a	21.49bc
		S2	4.21a	186abcd	140a	59.50a	22.00abc
		S3	4.28a	207a	112a	59.68a	21.30c
		Mean	3.68B	176B	127A	60.31B	21.80B
T3	P1	S1	0.81f	95e	71c	43.68c	18.47d
		S2	1.12ef	109de	89abc	52.13ab	18.61cd
		S3	1.28bcde	125bcd	90abc	50.64abc	19.69abcd
	P2	S1	1.18de	128bcd	99abc	45.95bc	19.94ab
		S2	1.51abcd	131bc	100abc	57.45a	19.17bcd
		S3	1.52abc	137bc	87bc	54.98a	20.02ab
	P3	S1	1.22cde	120cd	111ab	50.67abc	20.73a
		S2	1.43bcde	137abc	96abc	55.52a	20.34ab
		S3	1.44bcde	144ab	90abc	56.43a	20.25ab
	P4	S1	1.28bcde	129bcd	89abc	43.10c	19.75abc
		S2	1.55ab	140abc	103abc	58.23a	19.29bcd
		S3	1.84a	158a	121a	53.70ab	20.68a
		Mean	1.36C	129C	95B	51.92C	19.77C
**ANOVA**		T	***	***	***	***	***
		P	***	***	*	*	***
		S	***	***	ns	*	ns
		T * P	ns	ns	ns	ns	ns
		T * S	ns	ns	ns	***	ns
		P * S	ns	ns	ns	ns	ns
		T * P * S	ns	ns	ns	ns	ns

Different lowercase letters represent a significant difference at the maturity stage at 0.05 level according to the least significance difference (LSD) test. ***represents the significant difference at the 0.001 level according to the LSD test, **represents the significant difference at the 0.01 level according to the LSD test, *represents the significant difference at the 0.05 level according to the LSD test, and ns represents no significant difference. T1, T2, and T3 represent salinity levels of 0‰, 1.5‰, and 3‰, respectively. P1, P2, P3, and P4 represent no-priming treatment, ASA_160mg/L_ priming treatment, GABA_160mg/L_ priming treatment, and ZnO-Nano_200mg/L_ priming treatment, respectively. S1, S2, and S3 represent three sowing rates (90, 150, and 240 seeds m^−2^, respectively). The bold values and text represent the mean values of yield and its components under T1, T2, and T3 treatments. Different upper-case letters represent significant different at 0.05 probability level according to LSD.

**Table 3:** Effects of priming treatments and sowing rates on grain yield and its components in rice under salt stress in 2023 (average of two varieties).

Salinity	Priming treatment	Sowing rate	Yield (t ha^−1^)	Panicles (no. m^−2^)	Spikelets panicle^−1^	Filled grains (%)	1000-grain weight (g)
T1	P1	S1	6.19g	178g	170g	81.10d	24.24g
		S2	7.07f	196f	180fg	81.25d	24.50fg
		S3	7.59e	213e	185ef	83.30c	24.55fg
	P2	S1	7.71e	218e	176fg	83.98c	24.80ef
		S2	8.03d	242d	186ef	85.33b	24.85ef
		S3	8.72c	275b	193de	85.66b	25.45bcd
	P3	S1	7.54e	216e	193de	85.27b	25.08de
		S2	8.19d	220e	212b	85.24b	25.21bcde
		S3	8.62c	260c	217ab	86.96a	25.65b
	P4	S1	8.27d	232d	200cd	87.82a	25.11cde
		S2	9.27b	276b	210bc	87.38a	25.54bc
		S3	10.26a	308a	227a	87.74a	26.25a
		Mean	8.13A	236A	196A	85.09A	25.11A
T2	P1	S1	4.06g	111g	158g	81.36cd	19.58h
		S2	4.63f	134f	168defg	78.07e	20.07g
		S3	4.72f	155de	171cdef	78.30e	20.18fg
	P2	S1	4.67f	153e	166efg	81.35cd	20.16g
		S2	5.04de	169d	167efg	83.39ab	20.53f
		S3	5.80b	208b	179bcd	83.28abc	21.01e
	P3	S1	4.66f	153e	165fg	81.03d	21.63d
		S2	4.82ef	185c	173cdef	82.00bcd	21.79cd
		S3	5.48c	203b	180bc	82.11bcd	22.12bc
	P4	S1	5.09d	183c	176cde	81.78bcd	21.97cd
		S2	5.69bc	205b	187ab	84.57a	22.33b
		S3	6.40a	256a	196a	85.05a	22.95a
		Mean	5.09B	177B	174B	81.86B	21.20B
T3	P1	S1	1.55h	49h	96g	77.46de	16.33g
		S2	1.91g	79g	115ef	72.76g	17.03ef
		S3	2.17ef	97f	120def	73.24fg	17.10ef
	P2	S1	2.12ef	98f	104fg	76.28e	16.89ef
		S2	2.26de	121de	126cde	75.22efg	17.19ef
		S3	2.56bc	145b	142abc	76.08ef	18.01bc
	P3	S1	2.10ef	90fg	114ef	79.47cd	16.79fg
		S2	2.12ef	128cd	135bcd	82.62ab	17.36de
		S3	2.62b	151b	146ab	82.37ab	18.22b
	P4	S1	2.06fg	113e	119ef	80.58bc	17.72cd
		S2	2.40cd	139bc	143abc	84.11a	18.13bc
		S3	3.00a	168a	153a	83.35ab	19.41a
		Mean	2.24C	115C	126C	78.63C	17.51C
**ANOVA**		T	***	***	***	***	***
		P	***	***	***	***	***
		S	***	***	***	ns	***
		T * P	***	***	***	***	***
		T * S	***	ns	**	ns	**
		P * S	***	***	ns	***	***
		T * P * S	*	**	ns	***	ns

Different lowercase letters represent a significant difference at the maturity stage at 0.05 level according to the least significance difference (LSD) test. ***represents the significant difference at the 0.001 level according to the LSD test, **represents the significant difference at the 0.01 level according to the LSD test, *represents the significant difference at the 0.05 level according to the LSD test, and ns represents no significant difference. T1, T2, and T3 represent salinity levels of 0‰, 1.5‰, and 3‰, respectively. P1, P2, P3, and P4 represent no-priming treatment, ASA_160mg/L_ priming treatment, GABA_160mg/L_ priming treatment, and ZnO-Nano_200mg/L_ priming treatment, respectively. S1, S2, and S3 represent three sowing rates (90, 150, and 240 seeds m^−2^, respectively). The bold values and text represent the mean values of yield and its components under T1, T2, and T3 treatments. Different upper-case letters represent significant different at 0.05 probability level according to LSD.

### 3.6 Effects of priming and sowing rates on the K^+^/Na^+^

**Figure 8:**
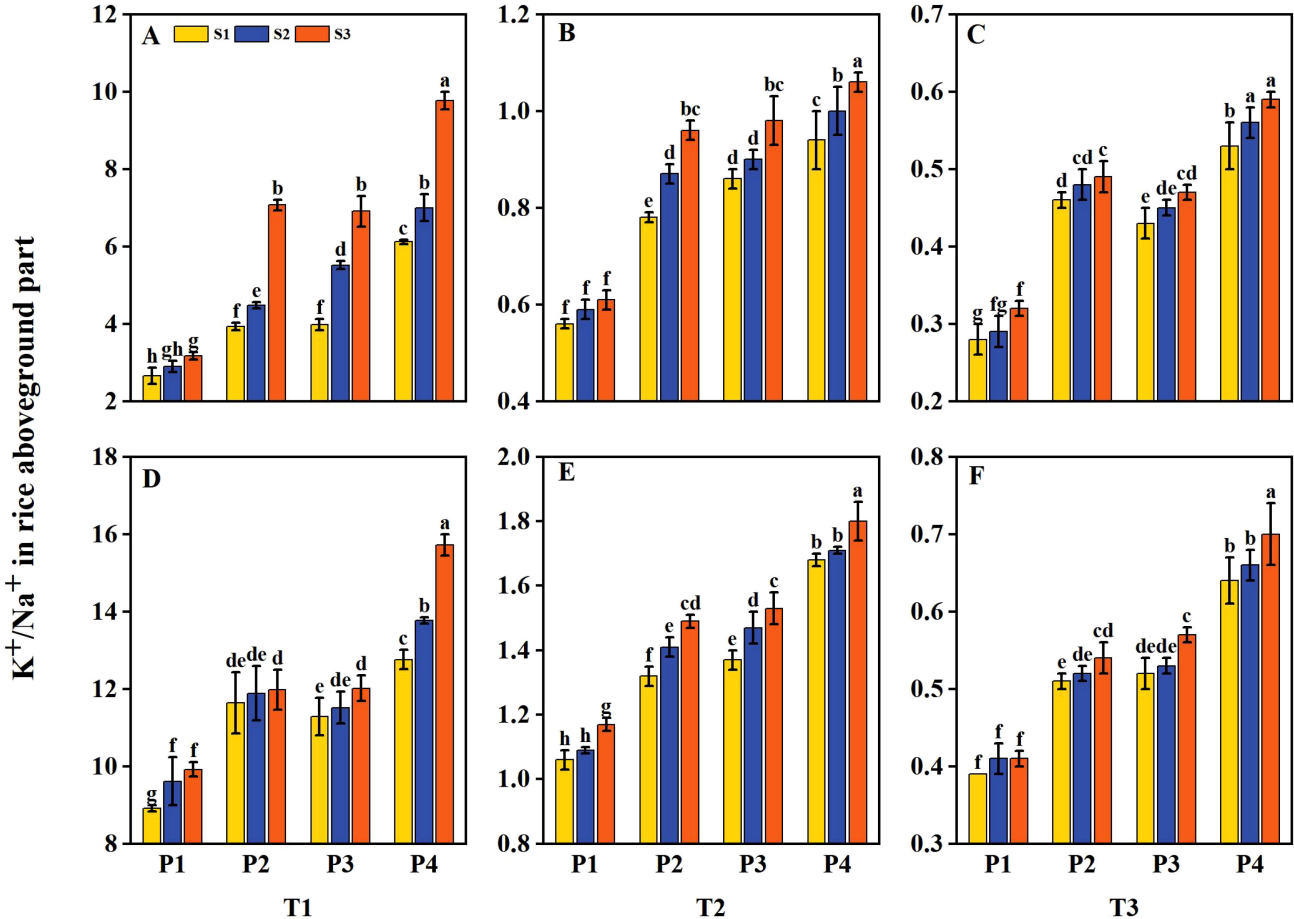
Effects of priming treatments and sowing rates on the K^+^/Na^+^ in rice aboveground part under salt stress in 2022 **(A–C)** and 2023 **(D–F)** (average of two varieties). Within a column, means followed by the same letter are not significantly different at the 0.05 probability level according to the least significant difference test (LSD 0.05). T1, T2, and T3 represent salinity levels of 0‰, 1.5‰, and 3‰, respectively. P1, P2, P3, and P4 represent no-priming treatment, ASA_160mg/L_ priming treatment, GABA_160mg/L_ priming treatment, and ZnO-Nano_200mg/L_ priming treatment, respectively. S1, S2, and S3 represent three sowing rates (90, 150, and 240 seeds m^−2^, respectively).

The aboveground Na^+^ and K^+^ contents of rice were influenced by salt stress and priming treatments. The K^+^/Na^+^ ratio in rice was decreased with increasing salt concentration (**Figure 8**). However, K^+^/Na^+^ was increased under priming and sowing rate treatments. Under T1, the K^+^/Na^+^ had average increases of 37.13%, 37.77%, and 75.14% under P2, P3, and P4 treatments, respectively, compared to P1 during 2 years. Under T2, the K^+^/Na^+^ had an average decrease of 86.76% compared to T1. Following P2, P3, and P4 treatments, the K^+^/Na^+^ had average increases of 34.52%, 40.48%, and 61.31%, respectively, compared to P1. Similarly, under T3, the K^+^/Na^+^ had an average decrease of 94.33% compared to T1, while after P2, P3, and P4 treatments, it had average increases of 43.48%, 42.03%, and 75.36%, respectively, compared to P1. Among all treatments, P4 consistently outperformed the other priming treatments under all salinity levels. Additionally, the K^+^/Na^+^ ratio increased with higher sowing rates under all salinity levels, with the sequence of changes at each priming treatment being S3 > S2 > S1. Moreover, across all salinity levels, the K^+^/Na^+^ ratio under the P2S1, P3S1, and P4S1 treatments was significantly higher than that under P1S3 during 2 years.

## 4 Discussion

### 4.1 The effect of seed priming on the germination rate and seedling physiology of DSR under salt stress

The seed germination rate of DSR under all priming treatments was significantly decreased under salt stress (T2 and T3) during 2 years (**Figure 2**). Liu et al. (2023) found that rice seed germination rate was closely linked to the structural integrity of cell membranes. The inhibition of germination under salt stress was primarily caused by plasma membrane damage and reduced amylase activity, leading to decreased or lost seed viability. ROS in seeds regulate cellular growth, protect against pathogens, and control redox status, playing a vital role in seed germination and seedling growth (Singh et al., 2016). However, under salt stress, the rapid accumulation of ROS induces oxidative damage to biomolecules, leading to necrosis and cell death (Abeed et al., 2023). According to our study, the decreased seed germination of DSR was caused by the decrease of α- amylase activity and soluble sugar and protein contents and the increase of ROS (O_2_^•−^ and H_2_O_2_) and MDA contents.

However, seed priming treatments could enhance seedlings’ emergence and stand establishment under salt stress. Seed priming initiates various germination-related activities, including early reserve mobilization and improved energy metabolisms (Adetunji, 2020). Furthermore, elevated levels of organic solutes, such as sugars and proteins, support plant metabolic adaptation, reduce cellular osmotic potential, and facilitate osmotic regulation under stress conditions (Matias et al., 2015). Seed priming treatments significantly increase sugars, proteins, α-amylase activity, and antioxidant system activity in seeds under abiotic stress (Sheteiwy et al., 2019; Sharma et al., 2021; Ibrahim, 2016). The process involves pre-exposure to salt stress, which augments the seeds’ resilience to subsequent stress conditions. Seed priming activates pre-germination metabolic pathways, enhancing physiological readiness for germination (Ibrahim, 2016). Our results demonstrated that under all three salinity levels, the seed germination rate of DSR was significantly improved after priming treatments. This enhancement was associated with elevated α-amylase activity, increased levels of soluble sugars and proteins, and reduced proline content, collectively improving seed vigor and salt stress tolerance. Under salt stress, ROS disrupts normal cellular metabolism by elevating MDA levels (Hasanuzzaman et al., 2021). Seed priming treatments activate ROS scavenging mechanisms (SOD, CAT, and POD), facilitating biochemical changes that enhance stress tolerance (Bussotti et al., 2014). The antioxidant system, comprising both enzymatic and non-enzymatic components, safeguards seeds by neutralizing ROS. A significant inverse correlation has been observed between antioxidant enzyme activity and MDA levels (Younesi and Moradi, 2014; Das et al., 2015). According to our study, the decreased ROS (O_2_^•−^ and H_2_O_2_) and MDA contents alongside increased activities of POD, SOD, and CAT ultimately improved the seed germination rate of DSR after priming treatments. These results align with the previous studies that confirmed ROS scavenging through activation of the antioxidant defense system (Ben Rejeb et al., 2014; Sedghi et al., 2010). Furthermore, our study also showed that under all three salinity levels, the seed germination rate was significantly higher under P4 compared to P2 and P3. This improvement was linked to elevated α-amylase activity, increased soluble sugar and protein contents, and enhanced antioxidant enzyme activities in rice seedlings. In recent years, the application of nanomaterials as catalysts to enhance seed stress resistance has gained significant attention (Khan et al., 2023). Studies have shown that seed nano-priming using nanoceria (CeO_2_ NPs) and silver nanoparticles (Ag NPs) elevates and sustains higher soluble protein levels in crops, thereby boosting metabolic processes. Other studies have indicated that nano-priming treatments facilitate greater water uptake during seed imbibition, further augmenting α-amylase activity and soluble sugar content, which collectively enhance seed germination under stress conditions (Mahakham et al., 2017). Moreover, Rai-Kalal and Jajoo (2021) demonstrated that faster water uptake, upregulation of aquaporin and hydrolytic transcript factors, and increased α-amylase activity to facilitate starch conversion into soluble sugars are key mechanisms of nano-priming. Our findings showed that under all three salinity levels, P4 treatment (ZnO-nanoparticle priming) achieved the highest germination rate among priming treatments. This was associated with higher α-amylase activity, increased soluble sugar and protein contents, and enhanced antioxidant enzyme activities in rice seedlings, aligning with findings from prior studies (Mu et al., 2023; Sultan et al., 2025). Therefore, seed priming technology especially the application of nano-priming, represents an effective strategy to enhance seedling emergence under salt stress conditions. Although nano-priming can promote crop growth, development, and yield, certain studies have reported that nanoparticles may inhibit crop growth, depending on their concentration and size (Mathur et al., 2023). Our research showed that a concentration of 200 mg L^−^¹ of ZnO nanoparticles did not inhibit crop development, consistent with the results of Liu et al. (2016), who reported that low concentrations (< 500 mg L^−^¹) of micronutrient metal oxide nanoparticles, such as ZnO, MnOx, and FeOx, improve seed germination and plant growth. Moreover, the complex interaction between nanoparticles, soil properties, and environmental factors appears to influence their impact on plants, warranting further investigation. Therefore, to mitigate nanoparticle phytotoxicity, future research should focus on stabilizing nanomaterials to maximize their potential (Phan and Haes, 2019).

### 4.2 Effects of priming and sowing rates on the plant growth and grain yield of DSR under salt stress

Grain yield was significantly reduced under salt stress, primarily attributed to declines in panicle number, spikelets per panicle, filled grain rate, and 1000-grain weight (Mumtaz et al., 2017; Gerona et al., 2019). In the present study, grain yield under elevated salinity levels (T2 and T3) was markedly reduced, primarily due to a decrease in panicle number (**Table 3**). Itroutwar et al. (2020) found that seed priming treatments can strengthen rice plants throughout the entire growth period. They also reported a strong correlation between rice yield and growth stage agronomic traits such as tiller number and aboveground biomass. Seed priming treatments could increase seedling density per unit area of crops under stress conditions, helping establish a strong seedling structure during the early stages of rice growth (Mondal and Bose, 2021). In this study, under all salinity levels, the seed germination rate was increased after priming treatments. This established a strong seedling structure, accelerated the tillering, and significantly increased the tillers (**Figure 6**) and panicle number per m^2^, thereby boosting rice yield. Moreover, the aboveground biomass of rice was increased (**Figure 7**) because seed priming promoted vegetative growth and enhanced stem length through cell division (Das et al., 2022).

An optimal seeding density can modulate rice population structure, mitigating the conflict between individual plant development and population growth (Cheng et al., 2012). Appropriately increasing the sowing rate can promote crop growth, enhance photosynthetic potential, and improve photosynthetically active radiation interception, which are crucial for the development of a dominant population (Jiang et al., 2021; Anjorin and Adebayo, 2024). Our results showed that under all three salinity levels, both aboveground biomass and tiller number increased with higher sowing rates under each priming treatment. Grain yield increased with higher sowing rates, primarily driven by a rise in panicle number. These findings align with those of Li et al. (2020), who reported that an increased sowing rate could boost tiller and panicle numbers and aboveground biomass, thereby enhancing the grain yield. In our study, under all three salinity conditions, the grain yield with priming treatment at 90 seeds m^−2^ was equivalent to that of rice without priming at 240 seeds m^−2^. Although increasing the sowing rate can improve rice emergence rate and grain yield, our study showed that reducing the sowing rate to 90 seeds m^−2^ with ZnO-nanoparticle priming treatment did not result in yield loss, significantly reducing cultivation costs in saline-alkali land. These results are consistent with the findings of Wang et al. (2014), who identified the optimal planting density for hybrid rice to be within the range of 70–118 seeds m^−2^. Therefore, the seed priming treatment could reduce the sowing rate under salt stress, thus lowering the cost of rice cultivation in saline-alkali soil. This cultivation measure provides practical feasibility for rice farming in saline areas.

Additionally, there was a substantial difference in average yields between the two years. The grain yield in 2023 (5.15 t ha^−1^) was 67.75% higher than that in 2022, mainly due to the increase in total solar radiation in 2023 (**Figure 1**), which significantly enhanced the light interception in the rice canopy, enhanced photosynthetic efficiency, and increased aboveground biomass, ultimately boosting yield (Lu et al., 2022). Furthermore, soil nutrient content significantly influenced yield. Studies have shown that soil nutrient levels affect nitrogen, phosphorus, and potassium absorption by plants, with a positive linear relationship observed between yield and the accumulation of these nutrients. Thus, differences in yield were primarily due to variations in soil fertility (Wang et al., 2015). Xiong et al. (2004) found that under the same fertilization and management conditions, soil with high organic matter content and elevated levels of one or two nutrients (N, P, or K) could increase yield, mainly due to the enhanced nutrient supply capacity of nutrient-rich soil. Other studies have shown that soil potassium has less effect on yield than nitrogen and phosphorus, and excessively high potassium levels can restrict the absorption of nitrogen and phosphorus in rice, thereby reducing yield (Yin et al., 2001). In the current study, notable variations in soil chemical properties were observed between the two seasons of the experimental field (**Table 1**). Therefore, the yield in 2023 was higher than that in 2022, mainly due to the higher nutrient content and stronger nutrient supply capacity of the 2023 experimental field. Additionally, the coordinated contents of N, P, and K in soil promoted nutrient absorption by rice, ultimately increasing yield.

### 4.3 Effects of priming and sowing rates on the K^+^/Na^+^ of rice

The Na^+^ content in rice straw increased significantly, while the K^+^ content and K^+^/Na^+^ ratio decreased under salt conditions. Salt stress inhibited plant growth primarily through ion and osmotic stress (Ran et al., 2023; Farooq et al., 2021). As salt stress increased, Na^+^ content was increased while K^+^ content decreased due to the antagonistic interaction between Na^+^ and K^+^, resulting in nutrient imbalance in rice plants (García et al., 2010). However, seed priming treatment could improve the physiological resilience of crops under salt stress, reducing ionic toxicity to plants and ultimately promoting growth (Khan et al., 2022; Biswas et al., 2023; Zulfiqar et al., 2022). Under salt stress, elevated activity of the K^+^ uptake system is a critical adaptive trait. The accumulation of K^+^ in plant cells plays a vital role in osmotic adjustment, aiding in the maintenance of cellular water balance. In guard cells, K^+^ is crucial for stomatal closure, thereby minimizing excessive water loss through transpiration. Recent studies have suggested that proteins regulating K^+^ uptake channels in guard cells may serve as potential targets for enhancing crop abiotic tolerance (Locascio et al., 2019). In the present study, seed priming and sowing rate treatments alleviated salt stress by significantly decreasing Na^+^ content and increasing K^+^ content and K^+^/Na^+^ ratio. Research has demonstrated that seed priming can reduce Na^+^ content while enhancing the uptake of K^+^ and Ca^+^ in crops, maintaining ion homeostasis of crops and thus enhancing crop salt tolerance (Farhoudi, 2011; Sheteiwy et al., 2021; Tan et al., 2022; Sen and Puthur, 2020). The underlying mechanism may involve priming treatments increasing K^+^ accumulation in rice, with adequate K^+^ levels promoting solute deposition, lowering osmotic potential, and enhancing turgor pressure under osmotic stress. Consequently, sufficient K^+^ concentration supported osmotic adjustment, resulting in increased turgor pressure, improved water uptake, and reduced osmotic potential. These physiological changes strengthened the plant’s capacity to withstand salt stress (Kumar et al., 2020). Moreover, our results showed that under all three salinity levels, both Na^+^ and K^+^ contents in rice tended to decrease with increasing sowing rate. The phenomenon may be attributed to increased competition for available resources at higher sowing rates, which reduced Na^+^ and K^+^ uptake (Santiago-Arenas et al., 2021). However, after priming treatment, the rice maintained higher α-amylase activity, soluble sugar content, and protein content, reducing K^+^ absorption loss and ultimately increasing the K^+^/Na^+^ ratio. Therefore, priming treatment can enhance the competitive advantage of K^+^ over other nutrients under high sowing rates through osmotic regulation, ultimately reducing the sowing rate while maintaining the balance of potassium and sodium at salt concentrations below 3‰.

## 5 Conclusion

The germination and growth of DSR were inhibited under salt stress, leading to reduced yield. Under salt stress conditions, the α-amylase activity, soluble sugar and protein contents, and the activities of SOD, POD, and CAT were increased by seed priming treatments at each sowing rate, improving seed germination and seedling growth, further contributing to an increase in panicle number and thus improving the grain yield. Under all three salinity conditions, the grain yield of DSR with seed priming at a sowing rate of 90 seeds m^−2^ was comparable to, or even exceeded, that of non-primed rice at 240 seeds m^−2^. This can be attributed to seed priming treatments, which promoted a reduction in cell osmotic potential and facilitated osmotic adjustment to the saline stress. Additionally, the increased activity of antioxidant enzymes helped scavenge ROS, thereby improving the germination rate, enhancing the tiller number, and increasing the K^+^/Na^+^ ratio. These physiological changes compensated for the challenges posed by the lower sowing rate under saline conditions. In addition, under each salinity level and sowing rate condition, the grain yield under the ZnO-nanoparticle priming treatment was always the highest. In conclusion, to ensure the economic benefits of DSR production when the salt concentration is below 3‰, the sowing rate of DSR could be reduced to 90 seeds m^−2^ using ZnO-nanoparticle priming treatment. Nevertheless, the molecular mechanisms underlying nano-priming-mediated enhancement of plant salt tolerance remain poorly understood. Therefore, further research is necessary to evaluate the technology on a broader range of rice varieties and assess its applicability under varying environmental conditions.

## Data Availability

The original contributions presented in the study are included in the article/**Supplementary Material**. Further inquiries can be directed to the corresponding author.
