# Supplementary material for: Regulation effect of seed priming on sowing rate of direct seeding of rice under salt stress
Source: Front Plant Sci. 2025 Mar 6;16:1541736. doi: 10.3389/fpls.2025.1541736 (PMC11922933; doi:10.3389/fpls.2025.1541736)
Supplement: Supplementary file 1 [file Image1.pdf]

Supplementary materials

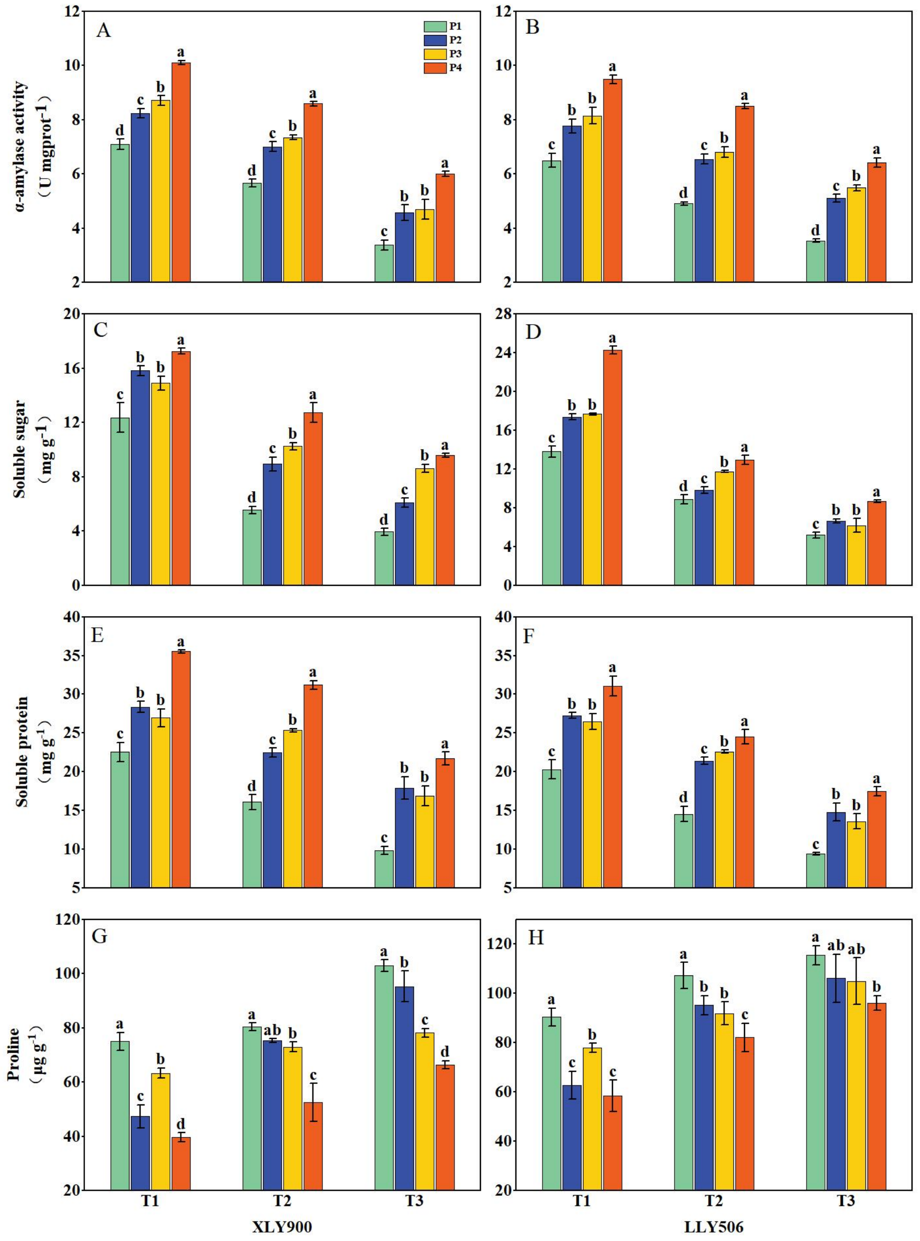

Figure S1 The α-amylase activity (A, B) and concentration of soluble sugar (C, D), soluble protein (E, F) and proline (G, H) of primed and non-primed seeds under different salt stress at 7 days after sowing. Note： Within a column, means followed by the same letter are not significantly different at the 0.05 probability level according to the least significant difference test (LSD 0.05). T1, T2, T3 were represent the salinity of 0‰, 1.5‰, 3‰; P1, P2, P3, P4 were represent no-priming treatment, $ASA_{160mg/L}$ priming treatment, $GABA_{160mg/L}$ priming treatment, and ZnO-$Nano_{200mg/L}$ priming treatment.

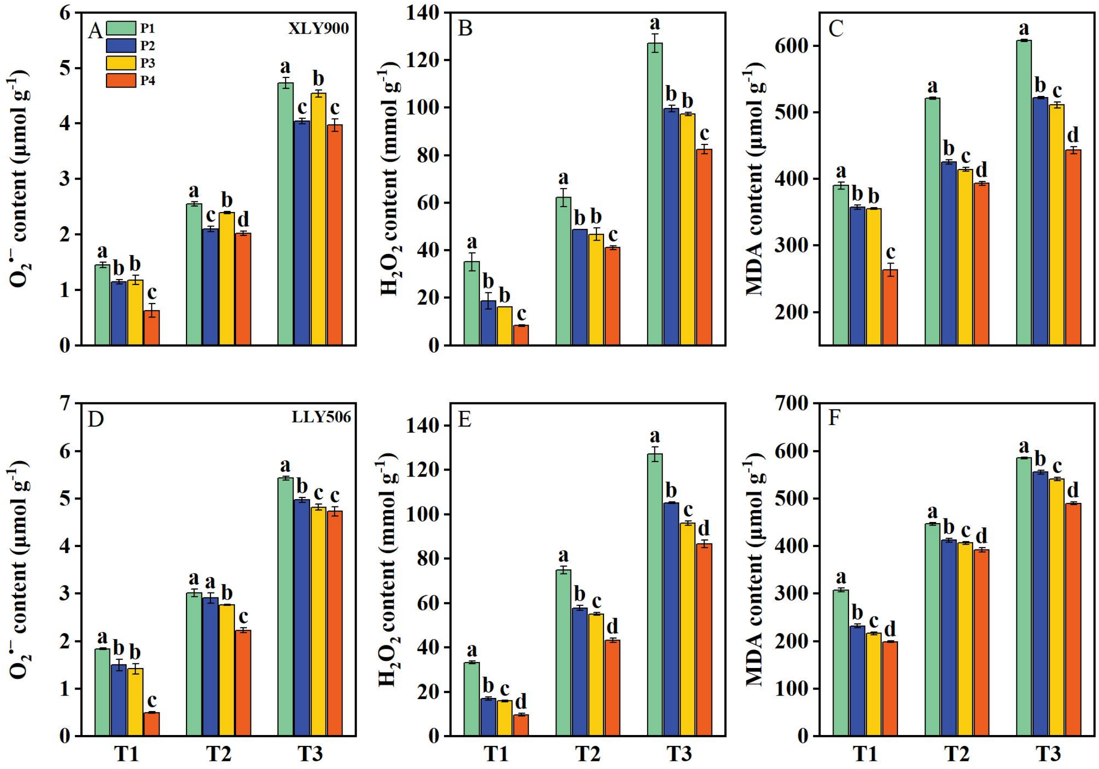

Figure S2 The concentration of $O_2^{\bullet -}$ (A, D), H_2O_2 (B, E) and MDA (C, F) of XLY900 (A, B, C) and LLY506 (D, E, F) under different salt stress at 7 days after sowing.

Note： Within a column, means followed by the same letter are not significantly different at the 0.05 probability level according to the least significant difference test (LSD 0.05). T1, T2, T3 were represent the salinity of 0‰, 1.5‰, 3‰; P1, P2, P3, P4 were represent no-priming treatment, $ASA_{160mg/L}$ priming treatment, $GABA_{160mg/L}$ priming treatment, and $ZnO\text{-}Nano_{200mg/L}$ priming treatment.

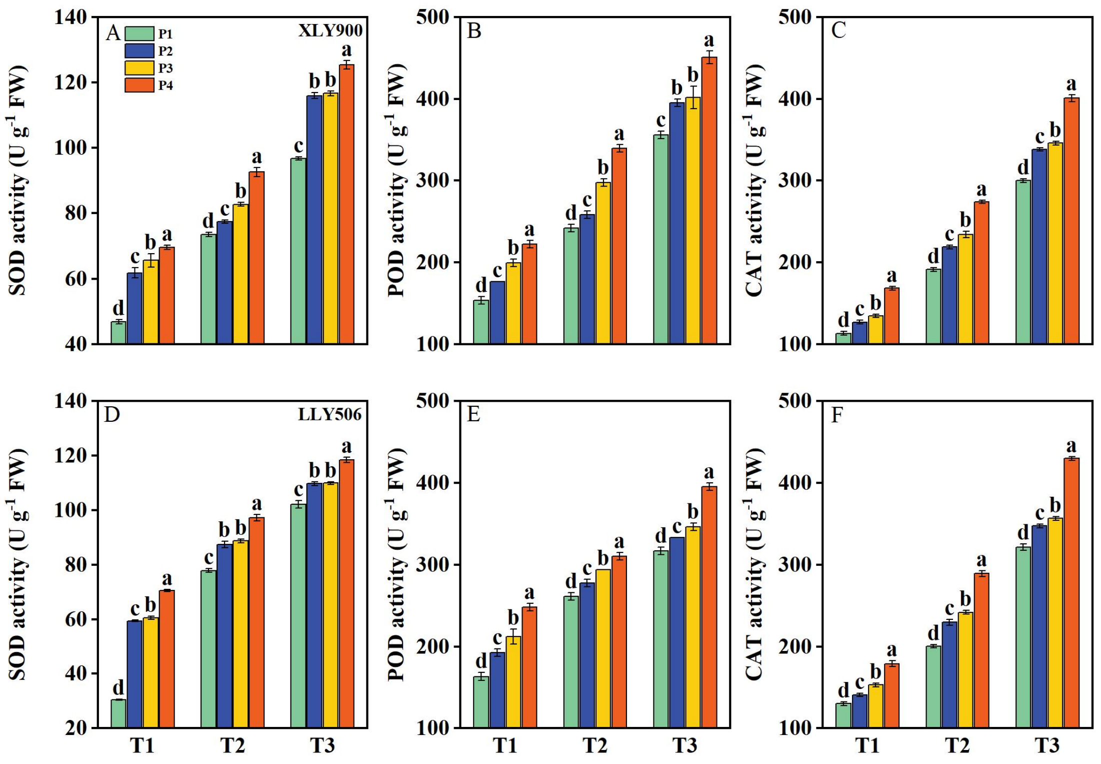

Figure S3 The activity of SOD (A, D), POD (B, E) and CAT (C, F) of XLY900 (A, B, C) and LLY506 (D, E, F) under different salt stress at 7 days after sowing.

Note： Within a column, means followed by the same letter are not significantly different at the 0.05 probability level according to the least significant difference test (LSD 0.05). T1, T2, T3 were represent the salinity of 0‰, 1.5‰, 3‰; P1, P2, P3, P4 were represent no-priming treatment, ASA$_{160mg/L}$ priming treatment, GABA$_{160mg/L}$ priming treatment, and ZnO-Nano$_{200mg/L}$ priming treatment.

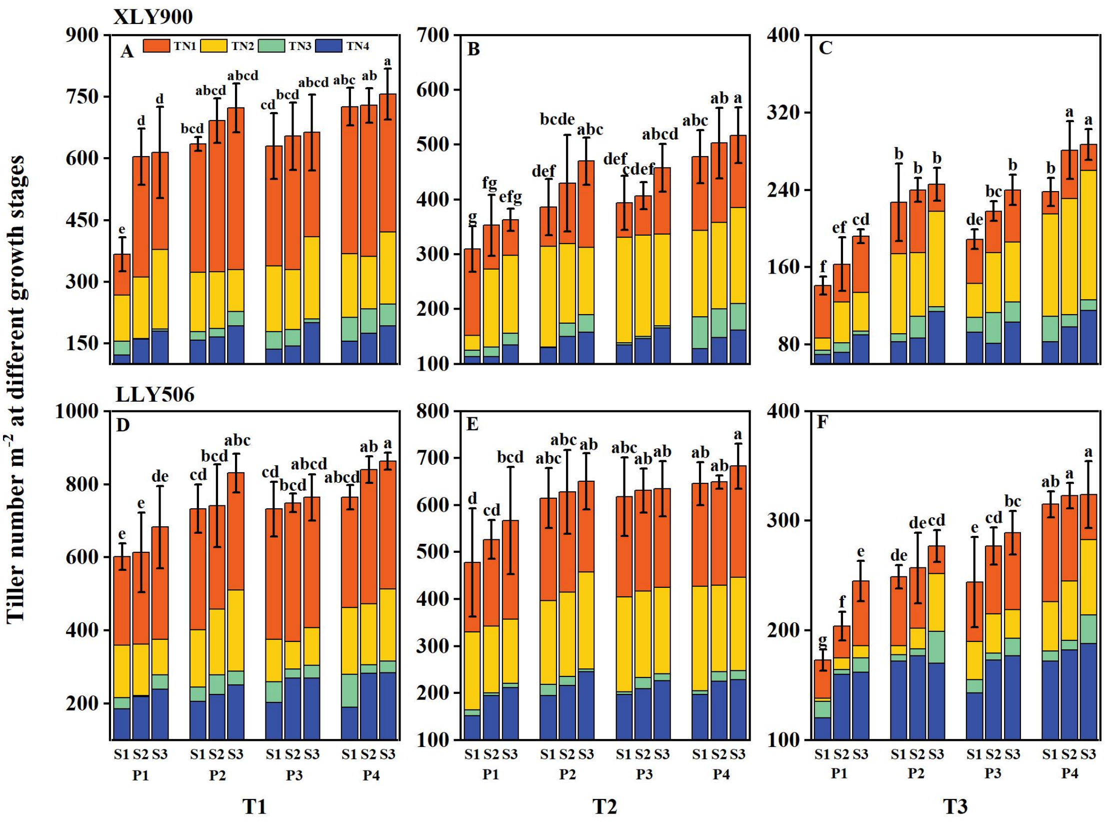

Figure S4 Effects of priming treatments and sowing rates on tiller number of XLY900 (A, B, C) and LLY506 (D, E, F) at different growth stages in 2022.

Note：Within a column, means followed by the same letter are not significantly different at the 0.05 probability level according to the least significant difference test (LSD 0.05). TN1 represents tiller number at the the mid-tillering stage; TN2 represents tiller number at the panicle initiation stage; TN3 represents tiller numbers at the heading stage; TN4 represents tiller number at the physiological maturity stage. T1, T2, T3 were represent the salinity of 0‰, 1.5‰, 3‰; P1, P2, P3, P4 were represent no-priming treatment, $ASA_{160mg/L}$ priming treatment, $GABA_{160mg/L}$ priming treatment, and $ZnO\text{-}Nano_{200mg/L}$ priming treatment; S1, S2, S3 were represent three sowing rates (90, 150, 240 seeds m^{-2}).

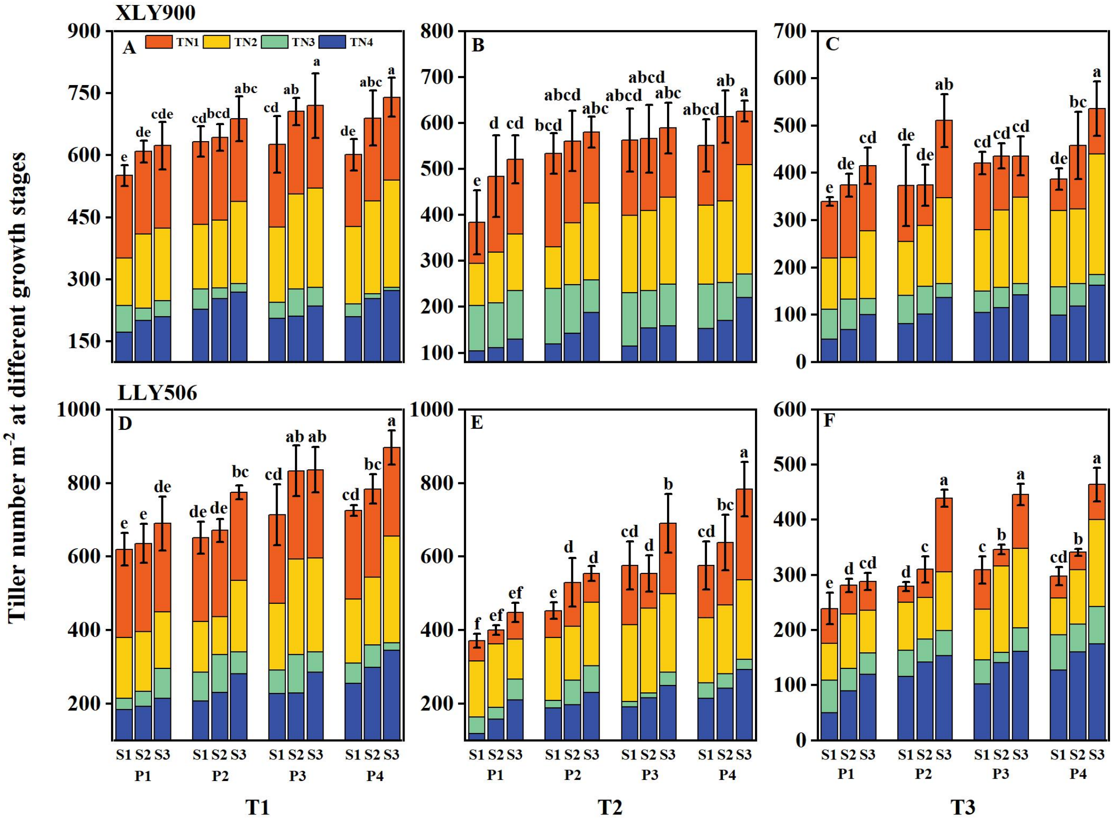

Figure S5 Effects of priming treatments and sowing rates on tiller number of XLY900 (A, B, C) and LLY506 (D, E, F) at different growth stages in 2023.

Note： Within a column, means followed by the same letter are not significantly different at the 0.05 probability level according to the least significant difference test (LSD 0.05). TN1 represents tiller number at the the mid-tillering stage; TN2 represents tiller number at the panicle initiation stage; TN3 represents tiller numbers at the heading stage; TN4 represents tiller number at the physiological maturity stage. T1, T2, T3 were represent the salinity of 0‰, 1.5‰, 3‰; P1, P2, P3, P4 were represent no-priming treatment, $ASA_{160mg/L}$ priming treatment, $GABA_{160mg/L}$ priming treatment, and $ZnO\text{-}Nano_{200mg/L}$ priming treatment; S1, S2, S3 were represent three sowing rates (90, 150, 240 seeds m^{-2}).

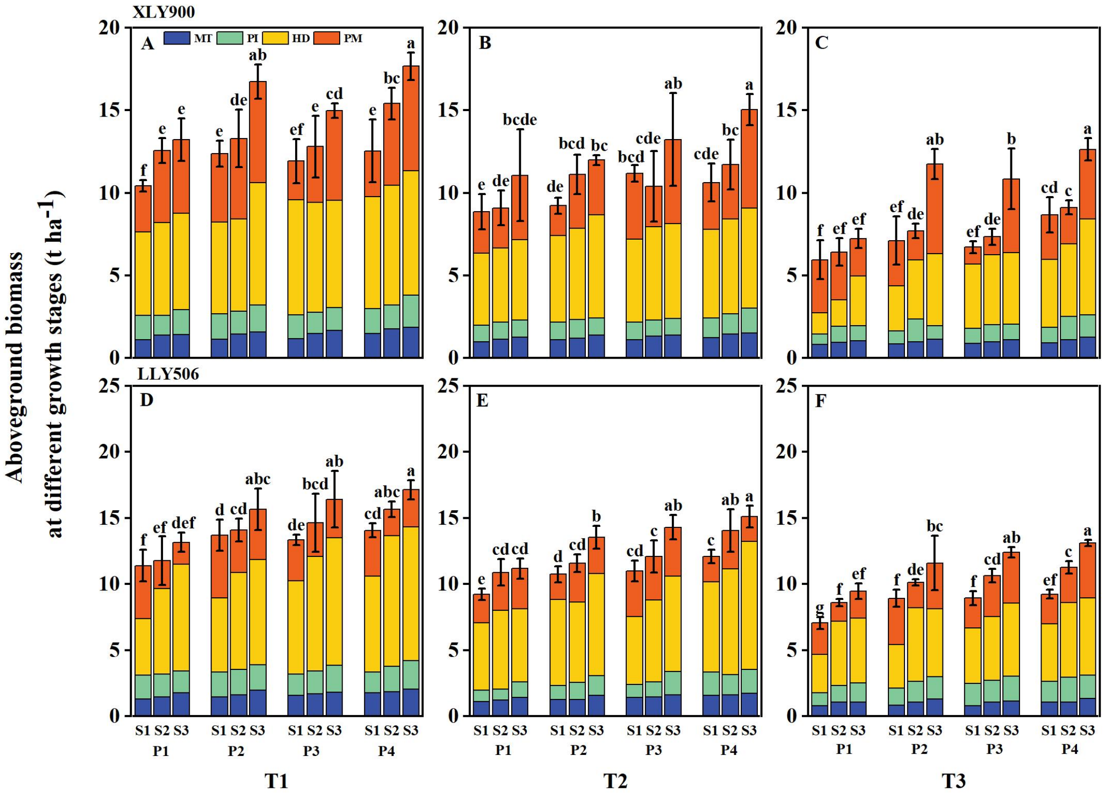

Figure S6 Effects of priming treatments and sowing rates on aboveground biomass of XLY900 (A, B, C) and LLY506 (D, E, F) at different growth stages in 2022.

Note：Different lower-case letters represent a significant difference at the maturity stage at 0.05 levels according to the LSD test. MT represents the mid-tillering stage; PI represents the panicle initiation stage; HD represents the heading stage; PM represents the physiological maturity stage. T1, T2, T3 were represent the salinity of 0‰, 1.5‰, 3‰; P1, P2, P3, P4 were represent no-priming treatment, $ASA_{160mg/L}$ priming treatment, $GABA_{160mg/L}$ priming treatment, and $ZnO\text{-}Nano_{200mg/L}$ priming treatment; S1, S2, S3 were represent three sowing rates (90, 150, 240 seeds m^{-2}).

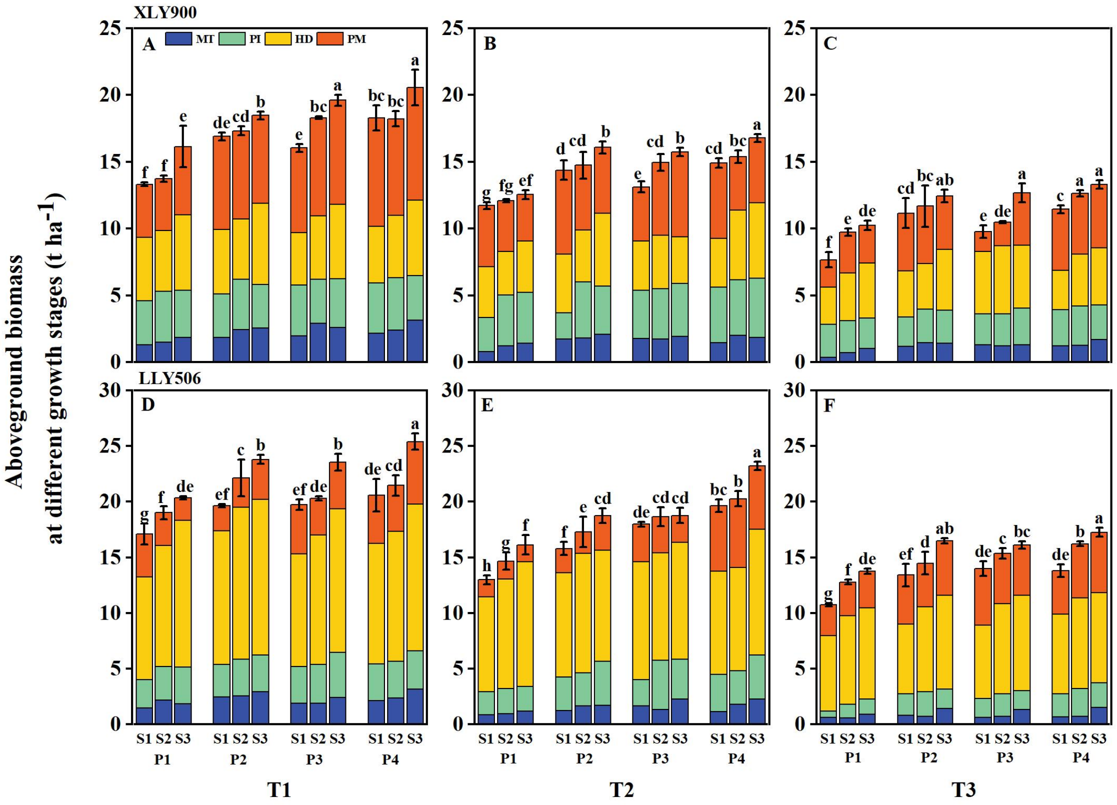

Figure S7 Effects of priming treatments and sowing rates on aboveground biomass of XLY900 (A, B, C) and LLY506 (D, E, F) at different growth stages in 2023.

Note：Different lower-case letters represent a significant difference at the maturity stage at 0.05 levels according to the LSD test. MT represents the mid-tillering stage; PI represents the panicle initiation stage; HD represents the heading stage; PM represents the physiological maturity stage. T1, T2, T3 were represent the salinity of 0‰, 1.5‰, 3‰; P1, P2, P3, P4 were represent no-priming treatment, $ASA_{160mg/L}$ priming treatment, $GABA_{160mg/L}$ priming treatment, and $ZnO\text{-}Nano_{200mg/L}$ priming treatment; S1, S2, S3 were represent three sowing rates (90, 150, 240 seeds m^{-2}).

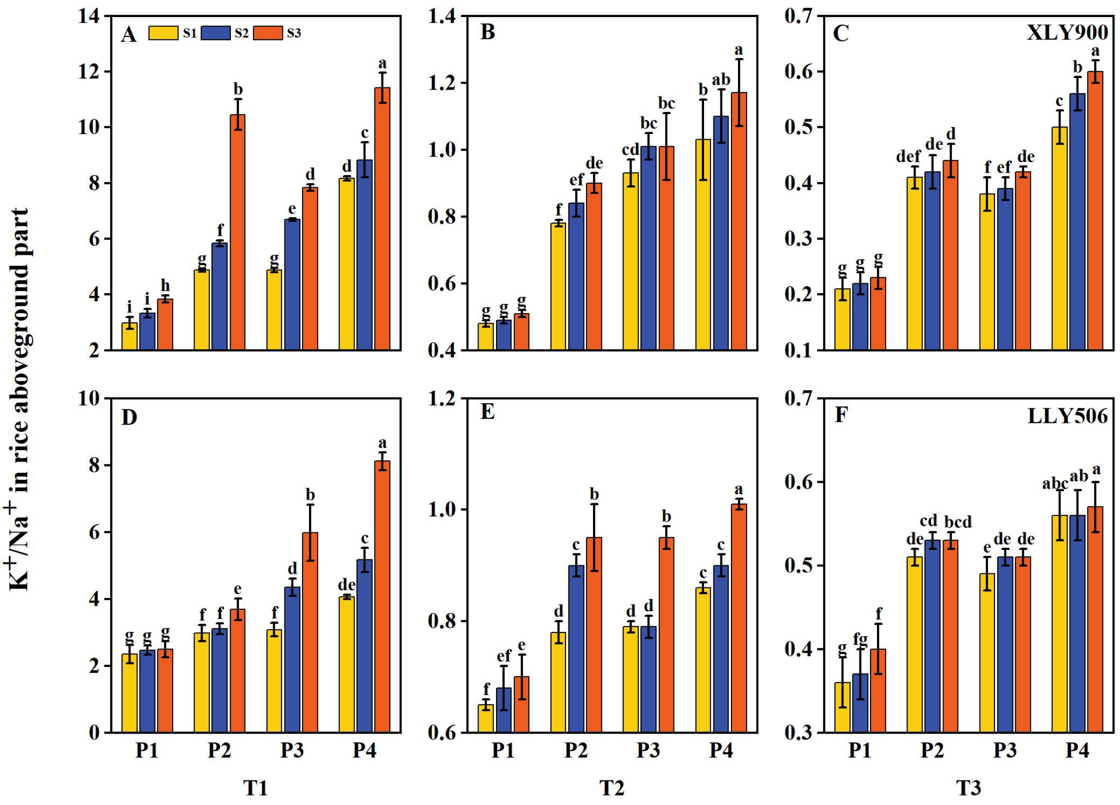

Figure S8 Effects of priming treatments and sowing rates on the K^+/Na^+ in rice aboveground part of XLY900 (A, B, C) and LLY506 (D, E, F) under salt stress in 2022.

Note： Within a column, means followed by the same letter are not significantly different at the 0.05 probability level according to the least significant difference test (LSD 0.05). T1, T2, T3 were represent the salinity of 0‰, 1.5‰, 3‰; P1, P2, P3, P4 were represent no-priming treatment, $ASA_{160mg/L}$ priming treatment, $GABA_{160mg/L}$ priming treatment, and $ZnO\text{-}Nano_{200mg/L}$ priming treatment; S1, S2, S3 were represent three sowing rates (90, 150, 240 seeds m^{-2}).

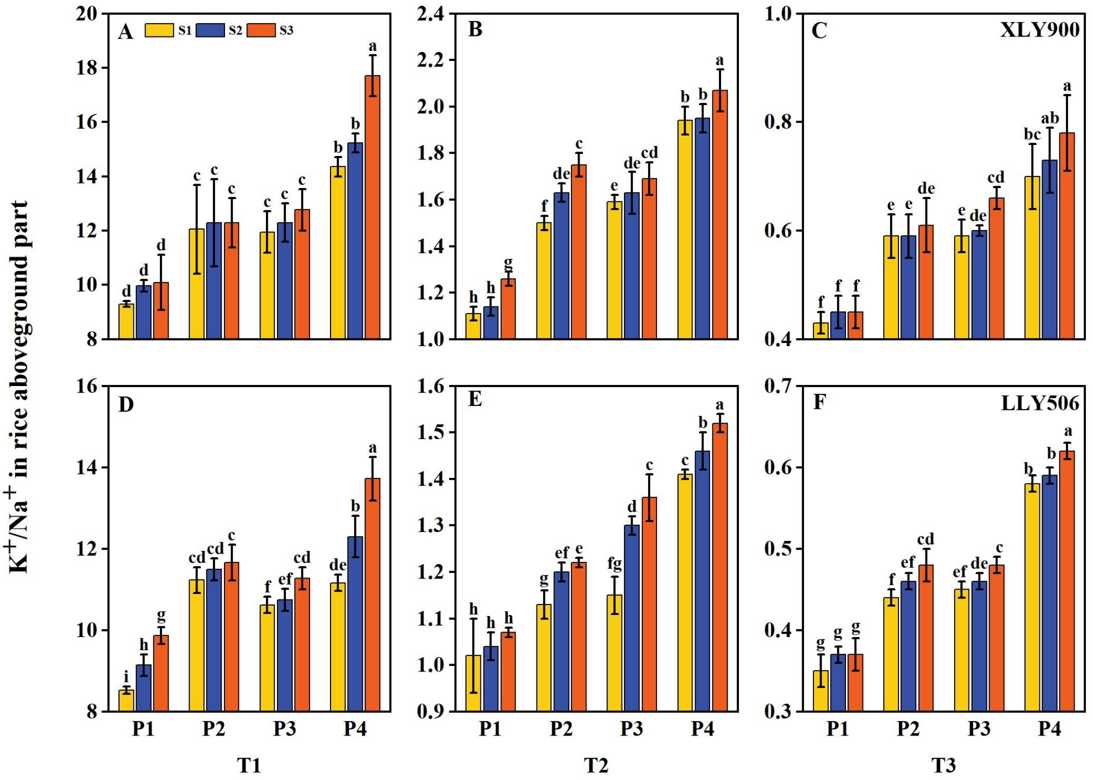

Figure S9 Effects of priming treatments and sowing rates on the K^+/Na^+ in rice aboveground part of XLY900 (A, B, C) and LLY506 (D, E, F) under salt stress in 2023.

Note：Within a column, means followed by the same letter are not significantly different at the 0.05 probability level according to the least significant difference test (LSD 0.05). T1, T2, T3 were represent the salinity of 0‰, 1.5‰, 3‰; P1, P2, P3, P4 were represent no-priming treatment, $ASA_{160mg/L}$ priming treatment, $GABA_{160mg/L}$ priming treatment, and ZnO-$Nano_{200mg/L}$ priming treatment; S1, S2, S3 were represent three sowing rates (90, 150, 240 seeds m^{-2}).
