# Supplementary material for: Regulation effect of seed priming on sowing rate of direct seeding of rice under salt stress
Source: Front Plant Sci. 2025 Mar 6;16:1541736. doi: 10.3389/fpls.2025.1541736 (PMC11922933; doi:10.3389/fpls.2025.1541736)
Supplement: Supplementary file 2 [file Table1.pdf]

Table S1 Effects of priming treatments and sowing rates on grain yield and its components in rice under salt stress in 2022.

Vareity	Salinity	Priming treatment	Sowing rate	Yield (t ha^{-1})	Panicles (no m^{-2})	Spikelets panicle^{-1}	Filled grains (%)	1000 grain weight (g)
V1	T1	P1	S1	3.87c	122c	152a	68.1ab	23.2b
			S2	4.08bc	160abc	152a	65.1ab	23.7ab
			S3	4.72abc	180ab	155a	69.6a	23.6ab
		P2	S1	4.05bc	158abc	159a	62.7ab	24.1a
			S2	4.67abc	165abc	156a	58.2b	23.9ab
			S3	5.62a	192a	156a	69.6a	24.2a
		P3	S1	4.21bc	143bc	171a	64.0ab	23.9ab
			S2	4.30bc	136bc	144a	67.5ab	24.3a
			S3	5.14abc	200a	178a	68.6ab	24.3a
		P4	S1	5.15ab	155abc	169a	63.3ab	24.0ab
			S2	4.68abc	174ab	149a	61.3ab	23.9ab
			S3	5.32ab	193a	158a	65.6ab	24.0ab
			**Mean**	**4.65A**	**164A**	**158A**	**65.3A**	**23.93A**
	T2	P1	S1	2.80c	113c	145a	62.9ab	21.8b
			S2	3.01c	113c	146a	62.0ab	22.0ab
			S3	3.60bc	135abc	143a	64.6ab	22.6ab
		P2	S1	3.59bc	130abc	153a	66.9ab	21.8b
			S2	3.68bc	150abc	148a	60.1b	22.4ab
			S3	4.20ab	158ab	143a	62.8ab	22.8ab
		P3	S1	3.81bc	135abc	166a	66.9ab	21.8ab
			S2	4.14ab	146abc	149a	65.2ab	22.9ab
			S3	4.49ab	128bc	164a	65.7ab	23.1a
		P4	S1	4.92a	166a	151a	68.9a	22.6ab
			S2	4.25ab	148abc	170a	62.6ab	23.2a
			S3	4.52ab	162ab	129a	65.0ab	22.7ab
			**Mean**	**3.92B**	**140B**	**151A**	**64.47A**	**22.47B**
	T3	P1	S1	0.91e	70b	73c	44.8c	19.6cd
			S2	1.05de	72b	90abc	51.8abc	18.6d
			S3	1.35bcde	90ab	99abc	50.1bc	21.2abc
		P2	S1	1.26cde	83ab	106abc	41.3c	20.3abcd
			S2	1.56abcd	87ab	107abc	58.6ab	19.6bcd
			S3	1.73abc	114a	89bc	53.2abc	21.0abc
		P3	S1	1.47bcde	93ab	132ab	53.5abc	21.5ab
			S2	1.52abcd	83ab	101abc	50.3bc	21.6ab
			S3	1.62abcd	103ab	97abc	57.3ab	21.6ab
		P4	S1	1.35cde	83ab	89bc	41.4c	20.9abc
			S2	1.95ab	98ab	113abc	63.4a	20.0bcd
			S3	2.04a	115a	149a	61.7ab	22.1a

			**Mean**	**1.50C**	**91C**	**103B**	**52.28B**	**20.67C**
V2	T1	P1	S1	3.12e	186d	98a	57.7a	21.5c
			S2	3.44bcde	218bcd	102a	56.8a	22.4bc
			S3	3.91abcd	239abcd	113a	59.8a	23.0ab
		P2	S1	3.44bcde	206cd	110a	60.7a	23.0ab
			S2	3.67bcde	225abcd	125a	63.2a	23.0ab
			S3	4.03abc	250abc	111a	60.5a	23.0ab
		P3	S1	3.19de	203cd	109a	59.6a	22.8ab
			S2	3.87abcde	269ab	113a	59.4a	23.1ab
			S3	4.09ab	269ab	119a	63.7a	23.4a
		P4	S1	3.31cde	189d	112a	58.3a	22.6ab
			S2	4.05ab	282a	112a	59.3a	22.5abc
			S3	4.60a	284a	114a	61.7a	22.7ab
			**Mean**	**3.73A**	**235A**	**112A**	**60.06A**	**22.75A**
	T2	P1	S1	2.98f	152d	94a	54.4a	20.9ab
			S2	3.01ef	194c	96a	54.0a	20.5b
			S3	3.25bcdef	211bc	99a	56.2a	20.8ab
		P2	S1	3.11def	195bc	109a	57.5a	21.4ab
			S2	3.47abcd	216abc	121a	56.5a	21.4ab
			S3	3.64a	228ab	109a	57.4a	20.9ab
		P3	S1	3.09def	197bc	106a	59.8a	21.5ab
			S2	3.38abcde	209bc	108a	58.1a	21.3ab
			S3	3.54abc	226ab	117a	57.2a	21.7a
		P4	S1	3.12cdef	197bc	111a	57.7a	20.7ab
			S2	3.63ab	225abc	119a	58.3a	21.2ab
			S3	3.76a	245a	101a	58.7a	21.3ab
			**Mean**	**3.33B**	**207B**	**108A**	**57.15B**	**21.13B**
	T3	P1	S1	0.71f	120d	81a	42.5d	17.8c
			S2	1.17cde	160bc	89a	52.5abcd	18.6abc
			S3	1.29bcd	162abc	81a	51.2abcd	18.2bc
		P2	S1	0.99def	172ab	93a	50.7abcd	19.6a
			S2	1.52abc	177ab	93a	56.3ab	18.7abc
			S3	1.64ab	170ab	81a	57.8a	19.0ab
		P3	S1	0.90ef	143cd	90a	47.8bcd	19.3a
			S2	1.43abc	173ab	90a	49.3abcd	19.1ab
			S3	1.55a	177ab	94a	52.9abc	19.1ab
		P4	S1	1.03def	172ab	90a	44.9cd	18.6abc
			S2	1.34abcd	182ab	93a	53.1abc	18.6abc
			S3	1.65a	188a	93a	57.9a	19.5a
			**Mean**	**1.27C**	**166C**	**89B**	**51.41C**	**18.84C**
		ANOVA	V	***	***	***	***	***
			T	***	***	***	***	***
			P	***	***	ns	ns	***
			S	***	***	ns	***	*

V×T	***	ns	**	**	*
V×P	*	ns	ns	ns	ns
V×S	ns	*	ns	ns	ns
T×P	ns	ns	ns	ns	ns
T×S	ns	*	ns	***	*
P×S	ns	ns	ns	ns	ns
V×T×P	ns	ns	ns	ns	ns
V×T×S	ns	ns	ns	ns	ns
V×P×S	ns	ns	ns	ns	ns
V×P×S	ns	ns	ns	ns	ns
V×T×P ×S	ns	ns	ns	ns	ns

Note： Different lower-case letters represent a signiicant difference at the maturity stage at 0.05 level according to the LSD test. *** represents the significant difference at the 0.001 level according to the LSD test, ** represents the significant difference at the 0.01 level according to the LSD test, * represents the significant difference at the 0.05 level according to the LSD test, and ns represents no significant difference. T1, T2, T3 were represent the salinity of 0‰, 1.5‰, 3‰; P1, P2, P3, P4 were represent no-priming treatment, $ASA_{160mg/L}$ priming treatment, $GABA_{160mg/L}$ priming treatment, and $ZnO\text{-}Nano_{200mg/L}$ priming treatment; S1, S2, S3 were represent three sowing rates (90, 150, 240 seeds m^{-2}).

Table S2 Effects of priming treatments and sowing rates on grain yield and its components in rice under salt stress in 2023.

Vareity	Salinity	Priming treatment	Sowing rate	Yield (t ha^{-1})	Panicles (no m^{-2})	Spikelets panicle^{-1}	Filled grains (%)	1000 grain weight (g)
V1	T1	P1	S1	7.05g	173f	170g	77.1f	25.82e
			S2	8.02ef	200e	183efg	82.7e	26.29de
			S3	8.25ef	210e	190def	78.2f	26.49de
		P2	S1	8.35de	228cd	178fg	86.28cd	26.79cd
			S2	8.71cd	254b	192de	83.45e	26.96bcd
			S3	9.61b	267ab	202cd	85.47cd	27.33abc
		P3	S1	7.84f	205e	212c	86.67bc	26.36de
			S2	8.92c	211de	229b	88.02ab	27.34abc
			S3	9.39b	235c	232ab	88.05a	27.6ab
		P4	S1	8.31de	210e	213c	86.53cd	26.69cd
			S2	9.51b	254b	226b	85.3d	26.99bcd
			S3	10.46a	273a	243a	85.57cd	28.01a
			**Mean**	**8.70A**	**227A**	**205A**	**84.45A**	**26.89A**
	T2	P1	S1	3.87f	104g	150e	83.65b	21.22g
			S2	4.7de	111fg	166cd	80.95de	21.85ef
			S3	5.06d	130ef	168cd	80.18e	21.53fg
		P2	S1	4.36e	119fg	162cde	85.87a	21.94ef
			S2	5.03d	143de	161de	81.6cde	22.32de
			S3	6.06b	188b	177bc	82.6bcd	22.87bc
		P3	S1	4.56e	115fg	159de	80.97de	22.92bc
			S2	4.97d	154cd	170cd	82.45bcd	23.07abc
			S3	5.78bc	159cd	173cd	81.33de	23.35ab
		P4	S1	5.44c	153cd	174cd	81.42de	22.71cd
			S2	6.14b	170bc	190ab	83.37bc	23.04bc
			S3	7.04a	220a	201a	83.52bc	23.6a
			**Mean**	**5.25B**	**147B**	**171B**	**82.32B**	**22.53B**
	T3	P1	S1	1.7g	49h	102e	79.82ab	17g
			S2	2.16de	69gh	132abc	76.38bc	17.84def
			S3	2.35cd	100de	135ab	73.35c	18.44cd
		P2	S1	1.96ef	81efg	113de	76bc	17.29efg
			S2	2.62ab	101de	132ab	73.48c	17.64efg
			S3	2.78a	136bc	136a	75.03c	18.63bcd
		P3	S1	1.91fg	78fg	113cde	75.45c	17.07fg
			S2	2.34cd	115cd	126abcd	83.07a	17.97de
			S3	2.77a	143ab	136a	80.73a	19.24ab
		P4	S1	2.13def	99def	117bcde	73.65c	18.51bcd
			S2	2.51bc	119cd	134ab	80.42a	18.91abc
			S3	2.87a	163a	139a	80.82a	19.69a
			**Mean**	**2.34C**	**104C**	**126C**	**77.35C**	**18.19C**

V2	T1	P1	S1	5.35h	184h	170e	85.12bc	22.68e
			S2	6.11g	193gh	178cde	83.92cd	22.72e
			S3	6.95f	215ef	180cde	84.3cd	22.61e
		P2	S1	7.08ef	208fg	174de	84.37cd	22.82e
			S2	7.37ef	230e	181cde	84.53cd	22.74e
			S3	7.83cd	281c	186bcde	85.85b	23.58bcd
		P3	S1	7.25ef	228e	175de	83.85d	23.81bc
			S2	7.47de	229e	196abc	82.45e	23.09de
			S3	7.86cd	285bc	203ab	85.87b	23.72bc
		P4	S1	8.25c	255d	187bcde	89.1a	23.55cd
			S2	9.05b	299b	194abcd	89.43a	24.1ab
			S3	10.08a	345a	212a	89.9a	24.5a
			**Mean**	**7.55A**	**246A**	**186A**	**85.72A**	**23.33A**
	T2	P1	S1	4.26h	119g	168d	79.08de	17.96i
			S2	4.58fgh	158f	172d	75.23f	18.29hi
			S3	4.9cdef	210cde	174cd	76.42ef	18.85fg
		P2	S1	4.74efg	188e	171d	76.83ef	18.39ghi
			S2	5.06cde	196de	175cd	85.18ab	18.76fgh
			S3	5.55ab	230bc	181abcd	83.95abc	19.16f
		P3	S1	4.45gh	191e	173cd	81.07cd	20.36e
			S2	4.67efgh	216cd	177bcd	81.53cd	20.51de
			S3	5.18bcd	249b	189ab	82.9bc	20.91cd
		P4	S1	4.75defg	215cd	180abcd	82.15c	21.25bc
			S2	5.26bc	241b	186abc	85.75ab	21.64b
			S3	5.77a	293a	193a	86.6a	22.32a
			**Mean**	**4.93B**	**209B**	**178A**	**81.39B**	**19.87B**
	T3	P1	S1	1.41e	50i	91d	75.05cd	15.67f
			S2	1.67de	90h	99cd	69.15e	16.21ef
			S3	1.99c	120fg	105cd	73.13de	16.88bcd
		P2	S1	1.63de	116fg	96cd	76.58cd	16.5de
			S2	1.9cd	143cde	121bc	76.95cd	16.74cde
			S3	2.36b	154bcd	148a	77.15c	17.39b
		P3	S1	1.6e	103gh	116cd	83.48b	16.51de
			S2	1.91cd	141de	146ab	82.15b	16.76cde
			S3	2.48b	161ab	156a	84.05ab	17.2bc
		P4	S1	1.99c	128ef	122bc	87.52a	16.95bcd
			S2	2.3b	160abc	153a	87.83a	17.36b
			S3	3.15a	175a	168a	85.9ab	19.14a
			**Mean**	**2.03C**	**128C**	**126B**	**79.91B**	**16.94C**
		ANOVA	V	***	***	*	***	***
			T	***	***	***	***	***
			P	***	***	***	***	***
			S	***	***	***	ns	***
			V×T	***	***	***	***	***

V×P	*	***	ns	***	***
V×S	***	*	ns	*	ns
T×P	***	***	***	***	***
T×S	***	ns	**	ns	***
P×S	***	*	ns	***	*
V×T×P	***	***	***	***	***
V×T×S	**	***	ns	***	ns
V×P×S	ns	ns	ns	***	ns
V×P×S	*	***	ns	***	ns
V×T×P×S	ns	*	ns	**	*

Note：Different lower-case letters represent a signiicant difference at the maturity stage at 0.05 level according to the LSD test. *** represents the significant difference at the 0.001 level according to the LSD test, ** represents the significant difference at the 0.01 level according to the LSD test, * represents the significant difference at the 0.05 level according to the LSD test, and ns represents no significant difference. T1, T2, T3 were represent the salinity of 0‰, 1.5‰, 3‰; P1, P2, P3, P4 were represent no-priming treatment, $ASA_{160mg/L}$ priming treatment, $GABA_{160mg/L}$ priming treatment, and ZnO-$Nano_{200mg/L}$ priming treatment; S1, S2, S3 were represent three sowing rates (90, 150, 240 seeds m^{-2}).
